# A robust ultrasensitive transcriptional switch in noisy cellular environments

**DOI:** 10.1038/s41540-024-00356-2

**Published:** 2024-03-16

**Authors:** Eui Min Jeong, Jae Kyoung Kim

**Affiliations:** 1 Biomedical Mathematics Group, Institute for Basic Science, 55, Expo-ro, Yuseong-gu, Daejeon, 34126 Republic of Korea; 2 Department of Mathematical Sciences, KAIST, 291, Daehak-ro, Yuseong-gu, Daejeon, 34141 Republic of Korea

**Keywords:** Cellular noise, Robustness

## Abstract

Ultrasensitive transcriptional switches enable sharp transitions between transcriptional on and off states and are essential for cells to respond to environmental cues with high fidelity. However, conventional switches, which rely on direct repressor-DNA binding, are extremely noise-sensitive, leading to unintended changes in gene expression. Here, through model simulations and analysis, we discovered that an alternative design combining three indirect transcriptional repression mechanisms, sequestration, blocking, and displacement, can generate a noise-resilient ultrasensitive switch. Although sequestration alone can generate an ultrasensitive switch, it remains sensitive to noise because the unintended transcriptional state induced by noise persists for long periods. However, by jointly utilizing blocking and displacement, these noise-induced transitions can be rapidly restored to the original transcriptional state. Because this transcriptional switch is effective in noisy cellular contexts, it goes beyond previous synthetic transcriptional switches, making it particularly valuable for robust synthetic system design. Our findings also provide insights into the evolution of robust ultrasensitive switches in cells. Specifically, the concurrent use of seemingly redundant indirect repression mechanisms in diverse biological systems appears to be a strategy to achieve noise-resilience of ultrasensitive switches.

## Introduction

Ultrasensitive responses in biological systems are input/output relationships that exhibit a highly sensitive response to changes in input or stimulus^1^. For example, transcriptional ultrasensitive switches are turned on or off depending on whether the level of the transcription factor exceeds or falls below a threshold. This distinctive property renders these switches valuable for systems requiring precise decision-making contingent on the number of input transcription factors. Moreover, these switches serve as catalysts for diverse cellular functions including signal amplification and generation of bistability and oscillations^2,3^, thereby bestowing them with versatile utility across various biological contexts. An ultrasensitive transcriptional switch can be generated by the cooperative binding of transcription factors to multiple DNA-binding sites^2–4^ or their titration via decoy sites^5^. And these mechanisms are used in the majority of synthetic transcriptional switches^6,7^. However, recent studies have raised concerns about the effectiveness of these conventional transcriptional switches in noisy cellular environments^8–10^. Specifically, when noise induces an undesired transition between the transcriptional ‘on’ and ‘off’ irrespective of the concentration of the transcription factor, this can persist for long periods.

Alternatively, transcriptional repressors can indirectly suppress transcription by targeting transcriptional activators rather than directly binding to DNA^11,12^. This suppression can occur by three different mechanisms. One mechanism is when repressors sequester transcriptional activators, preventing their binding to DNA. For example, sigma factors or basic leucine zippers can be sequestered through interactions with anti-sigma factors or inhibitors, respectively^13–15^. A second mechanism is when repressors bind to activators already bound to DNA, leading to transcriptional inhibition. This blocking is utilized when PHO80, a kinase component in a signaling pathway of yeast, inhibits PHO4^16^. In addition to sequestration and blocking, the third mechanism is when repressors displace DNA-bound activators. An illustrative case is the IκB protein, which reduces NF-κB activator’s binding affinity with DNA, displacing it from the DNA^17^. We recently found that the combination of these three mechanisms, sequestration, blocking, and displacement, can generate ultrasensitive transcriptional switches^18^. Interestingly, these three indirect repression mechanisms synergistically function in systems like the NF-κB oscillator and circadian clocks^17,19–23^, where precise ultrasensitivity against noise is crucial. This led us to hypothesize that an ultrasensitive switch based on the combination of multiple indirect repression mechanisms could be robust against noise unlike the conventional switches relying on direct repression.

Here, through model simulations and analysis, we found that the ultrasensitive switch based on the combination of the sequestration, blocking, and displacement can stably sustain the transcriptional ‘on’ and ‘off’ states even in the presence of noise by deriving the Fano factor. In this switch, the sequestration generates a sharp transition from the transcriptional ‘on’ state to the ‘off’ state as the molar ratio between repressors and activators shifts from below one to above one. However, even when the molar ratio surpasses and falls below one, transcriptional states can transit to the ‘on’ and ‘off’ states, respectively, due to noise. Then, the blocking and displacement immediately restore the original ‘off’ and ‘on’ states, respectively. This finding is supported by further analysis of identified mutation disrupting the displacement in the mammalian circadian clock. Taken together, we propose an ultrasensitive transcriptional switch that operates effectively even in noisy cellular environments and offers a promising strategy for designing robust synthetic switches.

## Results

### Noise triggers undesirable activation and repression of the cooperative binding-based switch

**Fig. 1:**
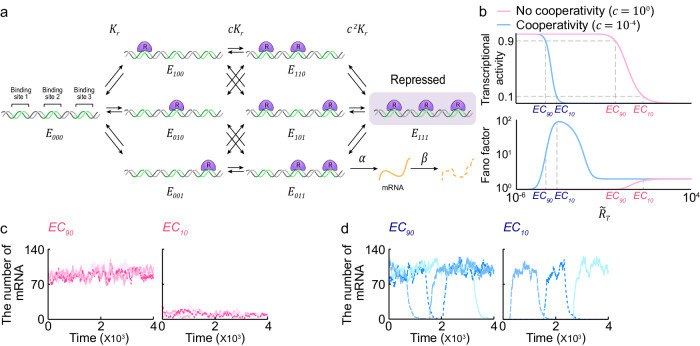
The transcriptional ultrasensitive switch based on cooperative binding suffers from undesired switch transition due to noise. **a** Schematic diagram of the model describing the binding of the repressor ($R$) to three sites on DNA. $R$ binds to an unoccupied site with a dissociation constant of ${K}_{r}$. When one site is occupied, $R$ binds to the other two sites with a dissociation constant of $c{K}_{r}$. When two sites are occupied, $R$ binds to the remaining site with a dissociation constant of ${c}^{2}{K}_{r}$. Accordingly, when $c \,<\, 1$, cooperativity is present, while absent when $c=1$. When all sites are occupied, the transcription is repressed (gray box); otherwise, mRNA ($M$) is transcribed with the rate of $\alpha$ and decays with the rate of $\beta$. **b** Consequently, as the total effective number of repressors ($\widetilde{{R}_{T}}$) increases, transcriptional activity decreases, and the system transitions from TAP to TRP (top). This transition in the presence of cooperativity (blue line) is more sensitive to changes in $\widetilde{{R}_{T}}$ compared to the absence of cooperativity (red line). On the other hand, Fano factor in the presence of cooperativity is higher compared to in the absence of cooperativity (bottom). **c** In the absence of cooperativity, the simulated timeseries of mRNA fluctuate around their high and low averages during TAP (i.e., when $\widetilde{{R}_{T}}$ is at $E{C}_{90}$) (left) and during TRP (i.e., when $\widetilde{{R}_{T}}$ is at $E{C}_{10}$) (right), respectively. **d** Conversely, in the presence of cooperativity, the number of mRNAs can significantly decrease and increase even during TAP (left) and TRP (right), respectively.

Ultrasensitivity can be generated through the cooperative binding of transcriptional repressors to multiple DNA sites^3,24^. To investigate this mechanism, we used a previously developed model of a cooperative binding-based switch^8^ (Fig. 1a and Supplementary Table 1). In this model, the DNA (${E}_{000}$) has three sites that can bind to the repressor ($R$) with a dissociation constant of ${K}_{r}$. When one site of the DNA is occupied (${E}_{001}$, ${E}_{010}$, or ${E}_{100}$), $R$ can bind to the other two sites with a dissociation constant of $c{K}_{r}$. Similarly, when two sites of the DNA are occupied (${E}_{110}$, ${E}_{101}$, or ${E}_{011}$), then $R$ can bind to the remaining site with a dissociation constant of ${c}^{2}{K}_{r}$. Therefore, when $c=1$, $R$ binds independently to each of the three sites, but when $c \,<\, 1$, $R$ binds more favorably to the DNA when one or two sites are already occupied than when all sites are unoccupied (i.e., $R$ cooperatively binds). Given that when all three sites are occupied by $R$ (${E}_{111}$), transcription is suppressed (Fig. 1a, gray box), the transcriptional activity is the probability that at least one site is unoccupied by $R$ at the steady state. We derived the probability with respect to the effective total number of the repressor $\widetilde{{R}_{T}}={R}_{T}/{K}_{r}$, where ${R}_{T}$ is the total number of repressors (Supplementary Note 1 and Supplementary Table 2).

The transcription is turned on with a high probability when $\widetilde{{R}_{T}}$ is low, referred to as the transcriptional activation phase (TAP; Fig. 1b, top). As $\widetilde{{R}_{T}}$ increases, the probability decreases to zero, reaching the transcriptional repression phase (TRP; Fig. 1b, top). The sharpness of this transition from transcriptional activation during TAP to repression during TRP depends on the cooperativity $c$. Specifically, when there is no cooperativity (i.e., $c=1$), the transition shows a similar sensitivity with the Michaelis-Menten equation (Fig. 1b, top, red line). In contrast, when repressors bind cooperatively (i.e., $c \,<\, 1$), the sensitivity of the transition increases (Fig. 1b, top, blue line). This sensitivity can be quantified using the effective Hill coefficient $\log 81/\log (E{C}_{10}/E{C}_{90})$, where $E{C}_{90}$ and $E{C}_{10}$ are the values of $\widetilde{{R}_{T}}$ at which the transcriptional activity becomes 0.9 and 0.1, respectively^1^. The measured effective Hill coefficients in the presence and absence of cooperativity are about 3 and 1.3, respectively (Fig. 1b, top).

Next, we found that, in the presence of cooperativity, transcriptional noise is higher than in the absence of cooperativity both at $E{C}_{90}$ for TAP and at $E{C}_{10}$, for TRP (Fig. 1b, bottom), as shown by the derived Fano factor of mRNA (Supplementary Note 1 and Supplementary Table 2). Specifically, in the absence of cooperative binding between repressors, mRNA numbers slightly fluctuate around their high average during TAP (Fig. 1c, left) and around their low average during TRP (Fig. 1c, right). However, in the presence of cooperativity, mRNA numbers often drop to zero even during TAP (Fig. 1d, left), and conversely often lift up to high levels even during TRP (Fig. 1d, right). These undesired transcriptional states, arising due to noise, persist because transition between the transcriptional activation and repression occurs rarely due to cooperative binding^8^. As a result, cooperativity increases the variance in the number of mRNAs transcribed, yielding a much higher Fano factor compared to the absence of cooperativity (Fig. 1b, bottom, and Supplementary Table 1).

### Noise turns on the sequestration-based switch during TRP

**Fig. 2:**
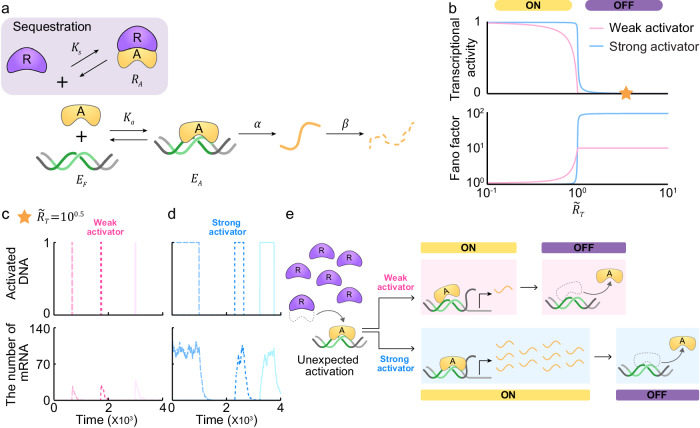
A transcriptional ultrasensitive switch based on solely sequestration is sensitive to noise during TRP due to undesired transcriptional activation. **a** Schematic diagram of the model describing the sequestration. Binding of the activator ($A$) to the DNA (${E}_{F}$) with a dissociation constant of ${K}_{a}$ forms the activated DNA (${E}_{A}$) to promote the transcription of mRNA ($M$) with the rate of $\alpha$, which decays with the rate of $\beta$. On the other hand, binding of the repressor ($R$) to $A$ with a dissociation constant of ${K}_{s}$ leads to transcriptional repression (gray box). **b** Consequently, when the molar ratio $\widetilde{{R}_{T}}={R}_{T}/{A}_{T}$ between the total activator (${A}_{T}$) and repressor (${R}_{T}$) is less than (TAP) and greater than one (TRP), transcription is turned on and off, respectively. Consequently, around $\widetilde{{R}_{T}}=1$, a sensitive transition from TAP to TRP occurs with respect to changes in $\widetilde{{R}_{T}}$. Notably, strong activators, which bind tightly to DNA, exhibit a more sensitive transition compared to weak activators binding loosely (top), while also showing higher transcriptional noise during TRP (bottom). **c**, **d** During TRP, the undesired activation is rapidly restored to the repressed state for a weak activator (**c**), but persists for a long period for a strong activator (**d**). **e** When noise-induced release of $A$ from the ${R}_{A}$ complex occurs, transcription can be turned on by binding of the $A$ to DNA. Such undesired activation during TRP persists for a long time with a strong activator due to tight binding (lower arrow), but not with a weak activator (upper arrow).

In the presence of sequestration, the free activator ($A$) binds to the free DNA (${E}_{F}$) with a dissociation constant of ${K}_{a}$ to form the activated DNA (${E}_{A}$). Transcription is inhibited when the free repressor ($R$) binds to $A$ to form an inactive complex (${R}_{A}$) with a dissociation constant of ${K}_{s}$ (i.e., sequestration; Fig. 2a, gray box, and Supplementary Table 1). Thus, the transcriptional activity is defined as the probability that the DNA is activated by $A$, not sequestered by $R$ at the steady state. We derived the probability as a function of $\widetilde{{R}_{T}}={R}_{T}/{A}_{T}$, the molar ratio between total numbers of the activator (${A}_{T}=A+{R}_{A}+{E}_{A}$) and the repressor (${R}_{T}=R+{R}_{A}$) (Supplementary Note 2 and Supplementary Table 2).

The transcription turns on and off depending on whether the molar ratio $\widetilde{{R}_{T}}$ is below or above one (Fig. 2b, top, and Supplementary Table 2). Specifically, when $\widetilde{{R}_{T}}$ is less than one (TAP), unsequestered activator promotes transcription. As the molar ratio exceeds one (TRP), a majority of activators become sequestered by repressors, promoting transcriptional repression. The transition between transcriptional activation during TAP and repression during TRP becomes more sensitive as the binding between $A$ and the DNA becomes stronger (i.e., $\widetilde{{K}_{a}}={K}_{a}/{A}_{T}$ decreases). This is because a strong activator (i.e., small $\widetilde{{K}_{a}}$) can promote transcription even when it is present in small numbers, sustaining transcriptional activation during TAP as long as there are more activators than repressors (i.e., $\widetilde{{R}_{T}} \,<\, 1$; Fig. 2b, blue line). However, when the molar ratio exceeds one (i.e., $\widetilde{{R}_{T}} > 1$; Fig. 2b), the majority of $A$ is sequestered by $R$. As a result, a sensitive transition from TAP to TRP occurs near $\widetilde{{R}_{T}}=1$ with the high effective Hill coefficient of about 50. On the other hand, the transition occurs gradually with the effective Hill coefficient of about 2 in the case of a weak activator (i.e., large $\widetilde{{K}_{a}}$; Fig. 2b, red line).

Next, we found that a strong activator (i.e., small $\widetilde{{K}_{a}}$; Fig. 2b, bottom, blue line) yields much higher transcriptional noise compared to a weak activator (i.e., large $\widetilde{{K}_{a}}$; Fig. 2b, bottom, red line) during TRP by deriving the Fano factor of mRNA (Supplementary Note 1 and Supplementary Table 1). For a weak activator, the activated DNA induced by noise is swiftly restored to the repressed state (Fig. 2c, top), keeping the number of mRNAs consistently low around zero (Fig. 2c, bottom). On the other hand, for a strong activator, this undesired activation of DNA during TRP persists over a long period (Fig. 2d, top), leading to a significant increase in the number of mRNAs (Fig. 2d, bottom). This disparity arises when a sequestered $A$ is released from$\,R$ and binds to the DNA despite the system being in the TRP (undesired activation; Fig. 2e). Subsequently, a weak activator would quickly dissociate from the DNA and thus transcription is turned off to its desired state (Fig. 2e, upper arrow). Meanwhile, a strong activator would remain bound to the DNA for a long time (Fig. 2e, lower arrow). As a result, the undesired transcriptional activation persists even during TRP, resulting in a considerable increase in the number of mRNAs. This leads to a bimodal distribution of mRNA molecules, resulting in high transcriptional noise, with a strong activator (Supplementary Fig. 1a, blue bars) unlike with a weak activator (Supplementary Fig. 1a, red bars). This bimodality arises due to noise despite the corresponding deterministic ODE model exhibits monostability^25^. In conclusion, when the binding between the activator and DNA is tight, a sensitive transcriptional response can be generated with sole sequestration. However, this tight binding leads to high transcriptional noise because undesired transcriptional activation during TRP persists for a long period.

### Noise turns off a sequestration- and blocking-based switch during TAP

**Fig. 3:**
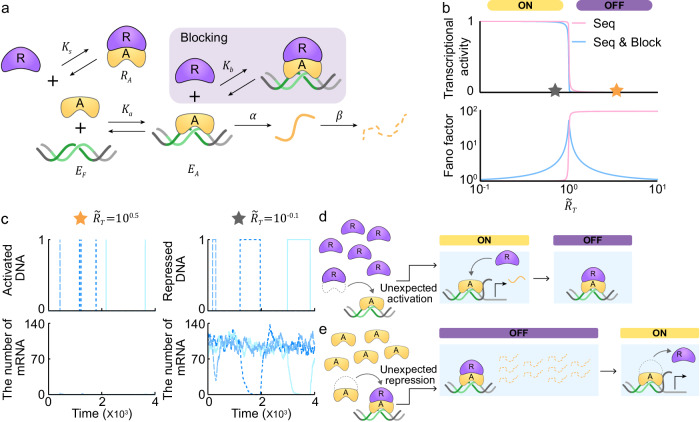
A transcriptional ultrasensitive switch based on the combination of sequestration and blocking is sensitive to noise during TAP due to undesired transcriptional repression. **a** A blocking mechanism is added to the sole sequestration model in Fig. 2a. The repressor ($R$) binds to the DNA-bound-activator (${E}_{A}$) with a dissociation constant of ${K}_{b}$ to block transcription (gray box). **b** The combination of sequestration and blocking (top, blue line) generates an ultrasensitive switch similar to sequestration alone (top, red line), but with reduced transcriptional noise during TRP and increased noise during TAP (bottom). **c** During TRP, the blocking rapidly restores the undesired activation of DNA to the repressed state (top left), and thus the number of mRNAs remains consistently near zero (bottom left). In contrast, during TAP, the undesired repression of DNA persists for long periods (top right), leading to a sharp decrease in mRNAs (bottom right). **d** When unwanted binding of activator to DNA occurs during TRP, the repressor rapidly inhibits the activator in the presence of blocking. **e** However, when noise-induced binding of repressor to DNA-bound-activator occurs during TAP, such undesired repression persists for a long time due to their tight binding.

When transcription is regulated by sequestration alone, high transcriptional noise occurs during TRP due to the absence of repression for the activator bound to the DNA. This DNA-bound activator can be suppressed when the repressor blocks transcription either by forming a complex with the DNA-bound-activator (i.e., blocking) or by pulling off the activator from the DNA (i.e., displacement). Indeed, these repression mechanisms are commonly employed in addition to the sequestration mechanism in many biological systems^11,18^. We first added blocking to the sole sequestration model, where the repressor ($R$) can directly bind to the DNA-bound-activator (${E}_{A}$) with a dissociation constant of ${K}_{b}$ to form the repressed DNA (${E}_{R}$; Fig. 3a, gray box, and Supplementary Table 1). The derived transcriptional activity exhibits a high sensitivity of response to changes in $\widetilde{{R}_{T}}$ similar to the sequestration (i.e., the effective Hill coefficient of about 50; Fig. 3b, top, and Supplementary Note 3). On the other hand, noise level is dramatically reduced during TRP (Fig. 3b, bottom, Supplementary Note 3 and Supplementary Table 1) as the addition of blocking rapidly restores the undesired activation of DNA to the repressed state (Fig. 3c, top left). This is evident in the simulated timeseries of mRNA, which remains close to zero (Fig. 3c, bottom left) without the sharp increase of mRNAs seen with sequestration alone (Fig. 2d, bottom). That is, the undesired transcription triggered by the noise-induced activator binding to DNA during TRP is immediately inhibited by the repressor via blocking (Fig. 3d) in contrast to sole sequestration (Fig. 2e, lower arrow). As a result, in the presence of blocking, the distribution of mRNA molecules is concentrated near zero (Supplementary Fig. 1b, blue bars) rather than bimodal (Supplementary Fig. 1b, red bars), reducing the transcriptional noise during TRP (Fig. 3b, bottom).

However, during TAP, the addition of blocking dramatically increases the transcriptional noise (Fig. 3b, bottom). With the blocking, the repression of DNA during TAP persists for a long period (Fig. 3c, top right), causing a dramatic reduction in the number of mRNAs (Fig. 3c, bottom right). This significant reduction arises when the repressor, released from the activator due to noise, binds to the DNA-bound activator during TAP (unexpected repression; Fig. 3e). Then, the repressor remains bound because there is no mechanism to release from the DNA-bound-activator until it will ultimately release itself (Fig. 3e). This causes a dramatic reduction in the number of mRNAs even during TAP, leading to a small additional peak around zero in the mRNA distribution (Supplementary Fig. 1c). Due to the bimodality, the transcriptional noise surges during TAP (Fig. 3b, bottom, and Supplementary Table 2).

### A sequestration-, blocking-, and displacement-based switch is robust to noise

**Fig. 4:**
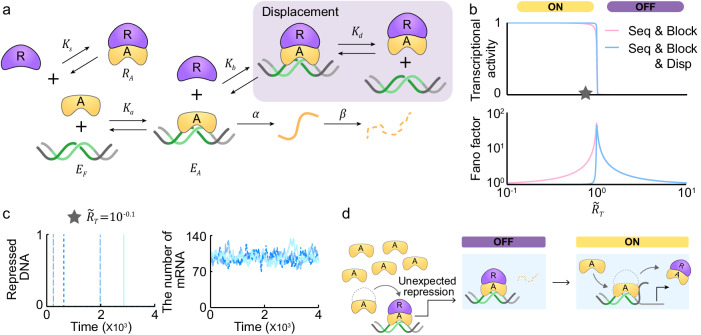
A transcriptional ultrasensitive switch based on the combination of sequestration, blocking, and displacement is robust to noise during both TAP and TRP. **a** Displacement is added to the blocking and sequestration model in Fig. 3a. The repressor-activator complex (${R}_{A}$) dissociates from DNA (${E}_{R}$) with a dissociation constant of ${K}_{d}$ to inhibit transcription (gray box). **b** The combination of sequestration, blocking, and displacement (top, blue line) generates an ultrasensitive switch similar to sequestration and blocking (top, red line), while reducing transcriptional noise during TAP (bottom). **c** With the addition of displacement, the undesired repression of DNA during TAP is rapidly restored to the activated state. **d** When unwanted repression occurs during TAP, the repressor-activator complex dissociates from DNA, and in the presence of displacement the free activator immediately promotes transcription.

In the presence of blocking, the undesired repression during TAP stems from the unwanted binding of the repressor to the activator on DNA. This could be prevented by the addition of a displacement mechanism, which facilitates dissociation of this repressor-activator complex from the DNA. To investigate this, we incorporated displacement into our blocking and sequestration model. In this model, the repressor-activator complex (${R}_{A}$) can dissociate from the DNA with a dissociation constant of ${K}_{d}$ (Fig. 4a, gray box, and Supplementary Table 1). The derived transcriptional activity can exhibit an ultrasensitive response to changes in $\widetilde{{R}_{T}}$ (Fig. 4b, top, Supplementary Note 4 and Supplementary Table 2). The transcriptional activity can exhibit an ultrasensitive response to changes in $\widetilde{{R}_{T}}$ with the effective Hill coefficient of about 200 (Fig. 4b, top).

Furthermore, the addition of displacement leads to a reduction in transcriptional noise during TAP (Fig. 4b, bottom, Supplementary Note 4 and Supplementary Table 2), as it rapidly restores the undesired repression of DNA to the activated state (Fig. 4c, left). This is evident in the simulated timeseries of mRNA, whose levels do not drop to zero (Fig. 4c, right) unlike with the combination of sequestration and blocking (Fig. 3c, bottom right). That is, when a repressor binds to the DNA-bound-activator due to noise during TAP (unexpected repression; Fig. 4d), the repressor-activator complex is immediately released from DNA via displacement (Fig. 4d). This does not happen without displacement (Fig. 3e). Taken together, the inclusion of displacement eliminates the additional peak near zero seen in the sequestration and blocking model (Supplementary Fig. 1d), leading to a reduction in transcriptional noise during TAP (Fig. 4b, bottom, and Supplementary Table 2).

This superiority of the triple-mechanism switch was established under the condition where the three types of switches exhibited different sensitivity of responses (Fig. 3b, top, and Fig. 4b, top). Thus, we modified the parameter value of $\widetilde{{K}_{b}}$ to make the mean mRNAs of the three types of switches the same, yielding the same sensitivity (Supplementary Fig. 2a, b). Even with the same sensitivity, the sequestration-, blocking-, and displacement-based switch shows lower mRNA variance compared to the two other switches (Supplementary Fig. 2c), resulting in a lower Fano factor and coefficient of variation (CV) of mRNAs (Supplementary Fig. 2d, e). So far, we used a fixed number of repressors when we investigated the transcriptional noise. To reflect the fluctuation of the total number of repressors in reality, we incorporated the birth and death of the repressor into the model^26^ (Supplementary Fig. 2f), leading to fluctuations in the number of repressors. Due to this fluctuation, all switches show reduced sensitivity in the means of mRNAs (Supplementary Fig. 2g), and furthermore exhibit increased variance of mRNAs (Supplementary Fig. 2h) compared to models without the fluctuation. As a result, the fluctuation in the number of repressors attenuates the robustness of all switches to noise (Supplementary Fig. 2i, j). However, even with this fluctuation, the triple-mechanism switch shows lower mRNA variance compared to the two other switches, while generating the same sensitivity (Supplementary Fig. 2g, h), leading to a lower Fano factor and CV of mRNAs (Supplementary Fig. 2i,j). Finally, to check whether the robustness of the switch combining the three mechanisms depends on the choice of parameters, we conducted a comprehensive analysis across a diverse parameter space. Specifically, we calculated the effective Hill coefficient and the area under the curve for the Fano factor during TAP and TRP, varying parameter values that represent the strength of each repression (i.e., $\widetilde{{K}_{a}}$, $\widetilde{{K}_{s}}$, $\widetilde{{K}_{b}}$, and $\widetilde{{K}_{d}}$; Supplementary Fig. 3). We found that the sequestration-, blocking-, and displacement-based switch can generate ultrasensitivity over a broader parameter range and is more resilient to noise than both the sole sequestration-based switch and the sequestration- and blocking-based switch (Supplementary Fig. 3).

## Discussion

Traditionally, ultrasensitive transcriptional responses are generated by cooperative binding of transcriptional repressors to multiple DNA sites. This mechanism can trigger a sharp transition from transcriptional repression during TRP to activation during TAP as the number of repressors surpasses a threshold. However, the presence of noise can lead to prolonged periods of undesired repression during TAP and undesired activation during TRP (Fig. 1). Thus, we shifted our focus from direct repression—where repressors directly bind to DNA, as seen in cooperative binding to multiple DNA sites—to the realm of indirect transcriptional repression: sequestration, blocking, and displacement. Sequestration alone can generate an ultrasensitive response that is attuned to changes in the molar ratio between repressors and activators. Specifically, when the number of repressors falls short of the number of activators (i.e., the molar ratio is less than one), an unsequestered activator binds to DNA, thereby promoting transcription. As the molar ratio surpasses one, a majority of activators become sequestered by repressors, promoting a sharp transition to transcriptional inhibition (Fig. 2). Furthermore, the transcriptional repression (activation) during TRP (TAP) can be stably maintained by blocking (displacement) even in the presence of noise. Specifically, when an activator that is unexpectedly released from repressors binds to DNA and initiates transcription during TRP (Fig. 2), the activator is immediately blocked by a repressor, resulting in the prompt restoration of transcriptional repression (Fig. 3). When a repressor unexpectedly blocks an activator that is already bound to DNA during TAP, the repressor-activator complex is displaced, swiftly reinitiating transcriptional activation (Fig. 4). Taken together, our exploration has unveiled an ultrasensitive transcriptional switch endowed with resilience against noise during both TAP and TRP.

Interestingly, although two models with or without a particular repression mechanism may appear similar in deterministic simulations, they can behave quite differently in stochastic simulations. Specifically, in the deterministic ODE, where the transcription of mRNA is proportional to the transcriptional activity, two models exhibiting similar transcriptional activity yield indistinguishable behaviors of mRNA^18^. On the other hand, in the stochastic models, the distributions of mRNA can be completely different despite resembling transcriptional activities. For example, even when all transcriptional activity of each model shows identical responses with respect to changes in the molar ratio (Supplementary Fig. 2b), whereas their Fano factors are completely different (Supplementary Fig. 2d). Therefore, a comprehensive understanding of the precise repression mechanism is essential to ensure unbiased modeling results when considering the presence of noise.

**Fig. 5:**
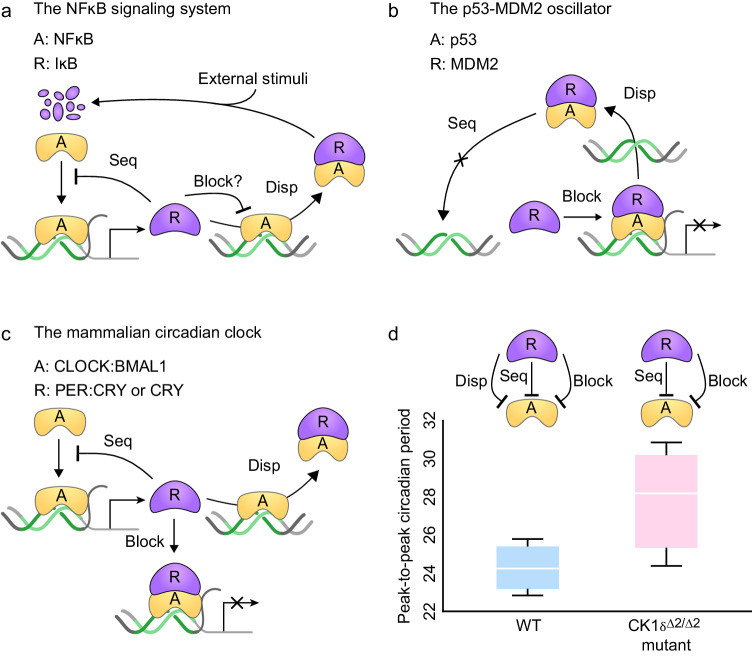
Various biological systems, requiring a precise ultrasensitive switch, utilize the combination of sequestration, blocking, and displacement. **a** In the NF-κB oscillator, IκB can repress stimulation-induced transcription by displacing the transcriptional activator NF-κB from DNA, as well as by sequestering it in the cytoplasm. It has not been investigated whether IκB can block the transcription by binding to DNA-bound-NF-κB. **b** In the p53-MDM2 oscillator, MDM2 binds with p53 to displace from DNA and then sequesters it not to bind with DNA. Furthermore, MDM2 can block the transcriptional activity of p53 with a corepressor. **c** In the mammalian circadian clock, the complex of PER and CRY inhibits their own transcription by sequestering and displacing the transcriptional activator CLOCK:BMAL1, while CRY inhibits the transcription by binding solely to the CLOCK:BMAL1:DNA complex. **d** When the displacement is disrupted by the CK1δ^Δ2/Δ2^ mutant in the mammalian circadian clock, the variance of peak-to-peak periods of circadian rhythms increases (WT: 24.0 ± 1.6 h, CK1δ^Δ2/Δ2^: 27.9 ± 3.6 h). The peak-to-peak period is measured using data retrieved from Etchegaray et al.

Biological oscillators and biological switches require ultrasensitivity to generate strong rhythms^27,28^ and bistable responses^2,29^, respectively. In addition to ultrasensitivity, these systems need to exhibit robustness against intrinsic noise to ensure accurate timing and precise decision-making. Indeed, such biological systems that require both ultrasensitivity and precision employ multiple indirect repression mechanisms. For instance, the NF-κB oscillator is known to utilize both sequestration and displacement to inhibit NF-κB by IκB^17,19^ (Fig. 5a). The p53-MDM2 oscillator^30,31^ (Fig. 5b), and circadian clocks in mammals (Fig. 5c)^21–23^ and *Drosophila*^20,32^ employ a combination of sequestration, blocking, and displacement. Notably, in the mammalian circadian clock, disruption of displacement by the CK1δ^Δ2/Δ2^ mutant^33^ leads to an increase in the variance of peak-to-peak periods of circadian rhythms (Fig. 5d). This result is consistent with our prediction that the combination of sequestration, blocking, and displacement is required for precise transcriptional on and off states in the mammalian circadian clock. However, as the CK1δ^Δ2/Δ2^ mutant can affect other components of the circadian clock beyond the displacement, it would be interesting in future work to investigate whether the increased variation solely results from disruption in the displacement. Furthermore, considering the frequent occurrence of feedback loops in the transcriptional switch, it would be intriguing to explore the robustness of the switch in the presence of feedback loops in future studies^27,28,34,35^.

Currently, existing synthetic switches have been designed based on direct DNA-binding mechanisms such as cooperative binding on multiple binding sites^36^, molecular titration via decoy binding sites^6^, and multistage transcriptional cascade^37^, or based on a single indirect repression mechanism, i.e., sequestration^38,39^. When these mechanisms generate ultrasensitivity, they become sensitive to noise. For instance, as super-enhancers activated via the cooperative binding of the transcriptional activator generate a more sensitive transcriptional response, the variance of mRNA distribution becomes larger^40^. Furthermore, as the binding affinity between the transcription factor and the decoy sites becomes stronger, the switch becomes more sensitive, but the noise level in transcription also increases^9^. Similarly, as the number of stages of cascades increases, the sensitivity of the switch increases, but the noise level in transcription also increases^37^. Furthermore, previous studies have also pointed out that a single sequestration-based switch can amplify noise^38^, consistent with our results (Fig. 2b, bottom). On the other hand, when additional indirect transcriptional repression mechanisms are added, while the sensitivity of transcriptional response increases, the sensitivity to noise decreases. Thus, the combination of the diverse indirect transcriptional repression mechanisms proposed in this study provides a design for synthetic ultrasensitive switches that are also robust to noise.

## Methods

We calculated the transcriptional activity and the Fano factor of mathematical models with an approach established by A. Sanchez et al.^8^. The equations resulting from this analysis are presented in Supplementary Table 2, while the detailed derivation process is provided in the Supplementary Information.

### Reporting summary

Further information on research design is available in the Nature Research Reporting Summary linked to this article.

### Supplementary information

Supplemental Information

Reporting Summary

## Data Availability

The datasets used and/or analyzed during the current study available from the corresponding author on reasonable request. The Julia codes to simulate the mathematical models describing each transcriptional switch are available at https://github.com/Mathbiomed/Ultrasensitive_Switch.
