# Supplementary material for: A robust ultrasensitive transcriptional switch in noisy cellular environments
Source: NPJ Syst Biol Appl. 2024 Mar 16;10:30. doi: 10.1038/s41540-024-00356-2 (PMC10944533; doi:10.1038/s41540-024-00356-2)
Supplement: Supplementary file 1 — Supplemental Information [file 41540_2024_356_MOESM1_ESM.pdf]

Supplementary Information

A robust ultrasensitive transcriptional switch in noisy cellular environments

Eui Min Jeong[1], and Jae Kyoung Kim[1,2]*
[1] Biomedical Mathematics Group, Institute for Basic Science, 55, Expo-ro, Yuseong-gu, Daejeon, 34126, Republic of Korea
[2] Department of Mathematical Sciences, KAIST, 291, Daehak-ro, Yuseong-gu, Daejeon, 34141, Republic of Korea
* To whom correspondence should be addressed. Tel: 82-42-350-2702; Email: jaekkim@kaist.ac.kr

Contents

Supplementary Note 1. The equations for the transcriptional activity and the Fano factor of the three binding sites model with cooperative binding.

Supplementary Note 2. The equations for the transcriptional activity and the Fano factor of the model describing the sequestration.

Supplementary Note 3. The equations for the transcriptional activity and the Fano factor of the model describing the sequestration, and blocking.

Supplementary Note 4. The equations for the transcriptional activity and the Fano factor of the model describing the sequestration, blocking, and displacement.

Supplementary Table 1. Propensity functions of reactions and parameter values for all models.

Supplementary Table 2. The transcriptional activity and the Fano factor for all models

Supplementary Figure 1. The stationary distributions of mRNAs transcribed during TRP and TAP in each transcriptional ultrasensitive switch

Supplementary Figure 2. The sequestration, blocking, and displacement-based switch exhibits superior robustness to noise compared to the other two types of switches, even when the means of the mRNAs are identical and the total number of repressors fluctuates

Supplementary Figure 3. The transcriptional switch utilizing all three mechanisms – sequestration, blocking and displacement – can generate ultrasenstivity robust to noise

Supplementary References

Supplementary Note 1. The equations for the transcriptional activity and the Fano factor of the three binding sites model with cooperative binding

We used an approach to calculate the transcriptional activity and the Fano factor is established by A. Sanchez et al[1]. Specifically, the transcription regulated by cooperative binding with three binding sites on DNA (Fig. 1a and Supplementary Table 1) can be described by the following CMEs:

$$\frac{dp_m}{dt} = Kp_m + \beta(m+1)p_{m+1} - \beta m p_m - Pp_m + Pp_{m-1}, \tag{1}$$

where

$$K = \begin{bmatrix} -3k_fR_T & k_r & k_r & k_r & 0 & 0 & 0 & 0 \\ k_fR_T & -2k_f(R_T-1)-k_r & 0 & 0 & ck_r & ck_r & 0 & 0 \\ k_fR_T & 0 & -2k_f(R_T-1)-k_r & 0 & ck_r & 0 & ck_r & 0 \\ k_fR_T & 0 & 0 & -2k_f(R_T-1)-k_r & 0 & ck_r & ck_r & 0 \\ 0 & k_f(R_T-1) & k_f(R_T-1) & 0 & -k_f(R_T-2)-2ck_r & 0 & 0 & c^2k_r \\ 0 & k_f(R_T-1) & 0 & k_f(R_T-1) & 0 & -k_f(R_T-2)-2ck_r & 0 & c^2k_r \\ 0 & 0 & k_f(R_T-1) & k_f(R_T-1) & 0 & 0 & -k_f(R_T-2)-2ck_r & c^2k_r \\ 0 & 0 & 0 & 0 & k_f(R_T-2) & k_f(R_T-2) & k_f(R_T-2) & -3c^2k_r \end{bmatrix},$$

$$P = \begin{bmatrix} \alpha & 0 & 0 & 0 & 0 & 0 & 0 & 0 \\ 0 & \alpha & 0 & 0 & 0 & 0 & 0 & 0 \\ 0 & 0 & \alpha & 0 & 0 & 0 & 0 & 0 \\ 0 & 0 & 0 & \alpha & 0 & 0 & 0 & 0 \\ 0 & 0 & 0 & 0 & \alpha & 0 & 0 & 0 \\ 0 & 0 & 0 & 0 & 0 & \alpha & 0 & 0 \\ 0 & 0 & 0 & 0 & 0 & 0 & \alpha & 0 \\ 0 & 0 & 0 & 0 & 0 & 0 & 0 & 0 \end{bmatrix}, \text{ and } p_m = \begin{bmatrix} p_m^{000} \\ p_m^{001} \\ p_m^{010} \\ p_m^{100} \\ p_m^{011} \\ p_m^{101} \\ p_m^{110} \\ p_m^{111} \end{bmatrix}.$$

Here, p_m is a probability vector whose components p_m^X represent the joint probability that DNA is in state E_X with m mRNAs, $X \in \{000, 001, 010, 100, 011, 101, 110, 111\}$. The stochastic transitions between DNA states are described by the matrix K, where R_T is the total number of repressors, k_f and k_r are the association and dissociation rates between repressors and DNA, respectively, and c is cooperativity. Additionally, P is the diagonal matrix whose diagonal elements represent the production rate of mRNA in each DNA state. Specifically, the last diagonal element of P is zero, because the transcription is suppressed only when all three binding sites are occupied by repressors (i.e., the state E_{111}). On the other hand, the remaining diagonal elements of P are α, which is the production rate of mRNA, as the transcription is activated in the remaining DNA states. Each mRNA is degraded with the

rate β. Hence, p_m decreases with the rate $\beta m p_m$ and increases with the rate $\beta(m+1)p_{m+1}$ in Eq. (1).

To reduce the number of parameters, we rescaled time variable t by $k_f R_T$ in Eq. (1) as follows:

$$\frac{dp_m}{d\tilde{t}} = \tilde{K}p_m + \beta(m+1)p_{m+1} - \beta m p_m - \tilde{P}p_m + \tilde{P}p_{m-1}, \tag{2}$$

where

$$\tilde{K} = \begin{bmatrix} -3 & \frac{1}{\widetilde{R_T}} & \frac{1}{\widetilde{R_T}} & \frac{1}{\widetilde{R_T}} & 0 & 0 & 0 & 0 \\ 1 & -2\left(1-\frac{1}{R_T}\right)-\frac{1}{\widetilde{R_T}} & 0 & 0 & \frac{c}{\widetilde{R_T}} & \frac{c}{\widetilde{R_T}} & 0 & 0 \\ 1 & 0 & -2\left(1-\frac{1}{R_T}\right)-\frac{1}{\widetilde{R_T}} & 0 & \frac{c}{\widetilde{R_T}} & 0 & \frac{c}{\widetilde{R_T}} & 0 \\ 1 & 0 & 0 & -2\left(1-\frac{1}{R_T}\right)-\frac{1}{\widetilde{R_T}} & 0 & \frac{c}{\widetilde{R_T}} & \frac{c}{\widetilde{R_T}} & 0 \\ 0 & 1-\frac{1}{R_T} & 1-\frac{1}{R_T} & 0 & -\left(1-\frac{2}{R_T}\right)-\frac{2c}{\widetilde{R_T}} & 0 & 0 & \frac{c^2}{\widetilde{R_T}} \\ 0 & 1-\frac{1}{R_T} & 0 & 1-\frac{1}{R_T} & 0 & -\left(1-\frac{2}{R_T}\right)-\frac{2c}{\widetilde{R_T}} & 0 & \frac{c^2}{\widetilde{R_T}} \\ 0 & 0 & 1-\frac{1}{R_T} & 1-\frac{1}{R_T} & 0 & 0 & -\left(1-\frac{2}{R_T}\right)-\frac{2c}{\widetilde{R_T}} & \frac{c^2}{\widetilde{R_T}} \\ 0 & 0 & 0 & 0 & 1-\frac{2}{R_T} & 1-\frac{2}{R_T} & 1-\frac{2}{R_T} & -\frac{3c^2}{\widetilde{R_T}} \end{bmatrix},$$

$$\tilde{P} = \begin{bmatrix} \tilde{\alpha} & 0 & 0 & 0 & 0 & 0 & 0 & 0 \\ 0 & \tilde{\alpha} & 0 & 0 & 0 & 0 & 0 & 0 \\ 0 & 0 & \tilde{\alpha} & 0 & 0 & 0 & 0 & 0 \\ 0 & 0 & 0 & \tilde{\alpha} & 0 & 0 & 0 & 0 \\ 0 & 0 & 0 & 0 & \tilde{\alpha} & 0 & 0 & 0 \\ 0 & 0 & 0 & 0 & 0 & \tilde{\alpha} & 0 & 0 \\ 0 & 0 & 0 & 0 & 0 & 0 & \tilde{\alpha} & 0 \\ 0 & 0 & 0 & 0 & 0 & 0 & 0 & 0 \end{bmatrix}, \text{ and } \tilde{t} = k_f R_T t.$$

Here, $K_r = k_r/k_f$ is the dissociation constant between repressors and free DNA, $\widetilde{R_T} = R_T/K_r$ is the effective total number of the repressor, $\tilde{\alpha} = \alpha/k_f R_T$ and $\tilde{\beta} = \beta/k_f R_T$ are normalized production and degradation rates of mRNA, respectively.

Then, from Eq. (2), we can calculate the ith moment vector of mRNAs at the steady state, $m_i = \sum_{m=0}^{\infty} m^i p_m$, whose elements represent the ith moment of mRNAs in each DNA state. First, the zeroth moment vector (i.e., the probability vector), m_0, can be calculated by summing p_m of Eq. (2) over m from 0 to infinity as follows:

$$\frac{dm_0}{d\tilde{t}} = \tilde{K}m_0 + \tilde{\beta}\sum_{m=0}^{\infty}(m+1)p_{m+1} - \tilde{\beta}\sum_{m=0}^{\infty} m p_m - \tilde{P}m_0 + \tilde{P}m_0 = \tilde{K}m_0. \tag{3}$$

Then, solving the steady state equation $0 = \tilde{K} m_0$ under the assumption that the number of binding sites is negligible compared to the total number of the repressor ($R_T \gg 3$), we can get

$$m_0 = \begin{bmatrix} \frac{c^3}{c^3+3c^3\widetilde{R_T}+3c^2\widetilde{R_T}^2\left(1-\frac{1}{R_T}\right)+\widetilde{R_T}^3\left(1-\frac{1}{R_T}\right)\left(1-\frac{2}{R_T}\right)} \\ \frac{c^3\widetilde{R_T}}{c^3+3c^3\widetilde{R_T}+3c^2\widetilde{R_T}^2\left(1-\frac{1}{R_T}\right)+\widetilde{R_T}^3\left(1-\frac{1}{R_T}\right)\left(1-\frac{2}{R_T}\right)} \\ \frac{c^3\widetilde{R_T}}{c^3+3c^3\widetilde{R_T}+3c^2\widetilde{R_T}^2\left(1-\frac{1}{R_T}\right)+\widetilde{R_T}^3\left(1-\frac{1}{R_T}\right)\left(1-\frac{2}{R_T}\right)} \\ \frac{c^3\widetilde{R_T}}{c^3+3c^3\widetilde{R_T}+3c^2\widetilde{R_T}^2\left(1-\frac{1}{R_T}\right)+\widetilde{R_T}^3\left(1-\frac{1}{R_T}\right)\left(1-\frac{2}{R_T}\right)} \\ \frac{c^2\widetilde{R_T}^2\left(1-\frac{1}{R_T}\right)}{c^3+3c^3\widetilde{R_T}+3c^2\widetilde{R_T}^2\left(1-\frac{1}{R_T}\right)+\widetilde{R_T}^3\left(1-\frac{1}{R_T}\right)\left(1-\frac{2}{R_T}\right)} \\ \frac{c^2\widetilde{R_T}^2\left(1-\frac{1}{R_T}\right)}{c^3+3c^3\widetilde{R_T}+3c^2\widetilde{R_T}^2\left(1-\frac{1}{R_T}\right)+\widetilde{R_T}^3\left(1-\frac{1}{R_T}\right)\left(1-\frac{2}{R_T}\right)} \\ \frac{c^2\widetilde{R_T}^2\left(1-\frac{1}{R_T}\right)}{c^3+3c^3\widetilde{R_T}+3c^2\widetilde{R_T}^2\left(1-\frac{1}{R_T}\right)+\widetilde{R_T}^3\left(1-\frac{1}{R_T}\right)\left(1-\frac{2}{R_T}\right)} \\ \frac{\widetilde{R_T}^3\left(1-\frac{1}{R_T}\right)\left(1-\frac{2}{R_T}\right)}{c^3+3c^3\widetilde{R_T}+3c^2\widetilde{R_T}^2\left(1-\frac{1}{R_T}\right)+\widetilde{R_T}^3\left(1-\frac{1}{R_T}\right)\left(1-\frac{2}{R_T}\right)} \end{bmatrix} \approx \begin{bmatrix} \frac{c^3}{c^3+3c^3\widetilde{R_T}+3c^2\widetilde{R_T}^2+\widetilde{R_T}^3} \\ \frac{c^3\widetilde{R_T}}{c^3+3c^3\widetilde{R_T}+3c^2\widetilde{R_T}^2+\widetilde{R_T}^3} \\ \frac{c^3\widetilde{R_T}}{c^3+3c^3\widetilde{R_T}+3c^2\widetilde{R_T}^2+\widetilde{R_T}^3} \\ \frac{c^3\widetilde{R_T}}{c^3+3c^3\widetilde{R_T}+3c^2\widetilde{R_T}^2+\widetilde{R_T}^3} \\ \frac{c^2\widetilde{R_T}^2}{c^3+3c^3\widetilde{R_T}+3c^2\widetilde{R_T}^2+\widetilde{R_T}^3} \\ \frac{c^2\widetilde{R_T}^2}{c^3+3c^3\widetilde{R_T}+3c^2\widetilde{R_T}^2+\widetilde{R_T}^3} \\ \frac{c^2\widetilde{R_T}^2}{c^3+3c^3\widetilde{R_T}+3c^2\widetilde{R_T}^2+\widetilde{R_T}^3} \\ \frac{\widetilde{R_T}^3}{c^3+3c^3\widetilde{R_T}+3c^2\widetilde{R_T}^2+\widetilde{R_T}^3} \end{bmatrix}. \quad (4)$$

The transcriptional activity, $TA(\widetilde{R_T})$, is defined as the probability that at least one binding site is unoccupied by repressor at the steady state. Thus, it can be derived by summing the first seven elements of m_0, which represent the probability that DNA is in the states with zero, one, or two repressors bound (Supplementary Table 2) as follows:

$$TA(\widetilde{R_T}) = 1 - \frac{\widetilde{R_T}^3}{c^3 + 3c^3\widetilde{R_T} + 3c^2\widetilde{R_T}^2 + \widetilde{R_T}^3}.$$

Similarly, multiplying both sides of Eq. (2) by m and m^2, and summing over m from zero to infinity, we can get equations for m_1 and m_2, respectively. Then, at the steady state,

$$\begin{aligned} 0 &= \tilde{K} m_1 + \tilde{\beta} \sum_{m=0}^{\infty} m(m+1) p_{m+1} - \tilde{\beta} \sum_{m=0}^{\infty} m^2 p_m - \tilde{P} \sum_{m=0}^{\infty} m p_m + \tilde{P} \sum_{m=0}^{\infty} m p_{m-1} \\ &= \tilde{K} m_1 + \tilde{\beta} \sum_{m=0}^{\infty} m(m-1) p_m - \tilde{\beta} \sum_{m=0}^{\infty} m^2 p_m - \tilde{P} \sum_{m=0}^{\infty} m p_m + \tilde{P} \sum_{m=0}^{\infty} (m+1) p_m \\ &= (\tilde{K} - \tilde{\beta} I) m_1 + \tilde{P} m_0, \end{aligned} \quad (5)$$

and

$$0 = \tilde{K} m_2 + \tilde{\beta} \sum_{m=0}^{\infty} m^2(m+1) p_{m+1} - \tilde{\beta} \sum_{m=0}^{\infty} m^3 p_m - \tilde{P} \sum_{m=0}^{\infty} m^2 p_m + \tilde{P} \sum_{m=0}^{\infty} m^2 p_{m-1} \quad (6)$$

$$= \tilde{K}m_2 + \tilde{\beta}\sum_{m=0}^{\infty}(m-1)^2 m p_m - \tilde{\beta}\sum_{m=0}^{\infty} m^3 p_m - \tilde{P}\sum_{m=0}^{\infty} m^2 p_m + \tilde{P}\sum_{m=0}^{\infty}(m+1)^2 p_m$$

$$= \tilde{K}m_2 - \tilde{\beta}\sum_{m=0}^{\infty}(2m^2 - m)p_m + \tilde{P}\sum_{m=0}^{\infty}(2m+1)p_m$$

$$= \left(\tilde{K} - 2\tilde{\beta}I\right)m_2 + \left(2P + \tilde{\beta}I\right)m_1 + \tilde{P}m_0,$$

where I is an identity matrix. As a result, solving Eq. (5) with the assumption $R_T \gg 3$, we can calculate the approximated m_1 as follows:

$$m_1 \approx \begin{bmatrix} \frac{\tilde{\alpha}}{\tilde{\beta}}\frac{c^3\left[(11+6\tilde{\beta}+\tilde{\beta}^2)\widetilde{R_T}^3+[3c^2(2+\tilde{\beta})(3+\tilde{\beta})+2c\tilde{\beta}(3+\tilde{\beta})+\tilde{\beta}(1+\tilde{\beta})]\widetilde{R_T}^2+c[6c^2(3+\tilde{\beta})+(3c+2)\tilde{\beta}]\widetilde{R_T}+6c^3\right]}{\widetilde{R_T}^3\left[(1+\tilde{\beta})(2+\tilde{\beta})(3+\tilde{\beta})\widetilde{R_T}^3+[3c^2(2+\tilde{\beta})(3+\tilde{\beta})+2c\tilde{\beta}(3+\tilde{\beta})+\tilde{\beta}(1+\tilde{\beta})]\widetilde{R_T}^2+c[6c^2(3+\tilde{\beta})+(3c+2)\tilde{\beta}]\widetilde{R_T}+6c^3\right]} \\ \frac{\tilde{\alpha}}{\tilde{\beta}}\frac{c^3\left[\tilde{\beta}(3+\tilde{\beta})^2\widetilde{R_T}^3+[3c^2(2+\tilde{\beta})(3+\tilde{\beta})+2c\tilde{\beta}(3+\tilde{\beta})+\tilde{\beta}(1+\tilde{\beta})]\widetilde{R_T}^2+c[6c^2(3+\tilde{\beta})+(3c+2)\tilde{\beta}]\widetilde{R_T}+6c^3\right]}{\widetilde{R_T}^2\left[(1+\tilde{\beta})(2+\tilde{\beta})(3+\tilde{\beta})\widetilde{R_T}^3+[3c^2(2+\tilde{\beta})(3+\tilde{\beta})+2c\tilde{\beta}(3+\tilde{\beta})+\tilde{\beta}(1+\tilde{\beta})]\widetilde{R_T}^2+c[6c^2(3+\tilde{\beta})+(3c+2)\tilde{\beta}]\widetilde{R_T}+6c^3\right]} \\ \frac{\tilde{\alpha}}{\tilde{\beta}}\frac{c^3\left[\tilde{\beta}(3+\tilde{\beta})^2\widetilde{R_T}^3+[3c^2(2+\tilde{\beta})(3+\tilde{\beta})+2c\tilde{\beta}(3+\tilde{\beta})+\tilde{\beta}(1+\tilde{\beta})]\widetilde{R_T}^2+c[6c^2(3+\tilde{\beta})+(3c+2)\tilde{\beta}]\widetilde{R_T}+6c^3\right]}{\widetilde{R_T}^2\left[(1+\tilde{\beta})(2+\tilde{\beta})(3+\tilde{\beta})\widetilde{R_T}^3+[3c^2(2+\tilde{\beta})(3+\tilde{\beta})+2c\tilde{\beta}(3+\tilde{\beta})+\tilde{\beta}(1+\tilde{\beta})]\widetilde{R_T}^2+c[6c^2(3+\tilde{\beta})+(3c+2)\tilde{\beta}]\widetilde{R_T}+6c^3\right]} \\ \frac{\tilde{\alpha}}{\tilde{\beta}}\frac{c^3\left[\tilde{\beta}(3+\tilde{\beta})^2\widetilde{R_T}^3+[3c^2(2+\tilde{\beta})(3+\tilde{\beta})+2c\tilde{\beta}(3+\tilde{\beta})+\tilde{\beta}(1+\tilde{\beta})]\widetilde{R_T}^2+c[6c^2(3+\tilde{\beta})+(3c+2)\tilde{\beta}]\widetilde{R_T}+6c^3\right]}{\widetilde{R_T}^2\left[(1+\tilde{\beta})(2+\tilde{\beta})(3+\tilde{\beta})\widetilde{R_T}^3+[3c^2(2+\tilde{\beta})(3+\tilde{\beta})+2c\tilde{\beta}(3+\tilde{\beta})+\tilde{\beta}(1+\tilde{\beta})]\widetilde{R_T}^2+c[6c^2(3+\tilde{\beta})+(3c+2)\tilde{\beta}]\widetilde{R_T}+6c^3\right]} \\ \frac{\tilde{\alpha}}{\tilde{\beta}}\frac{c^2\left[\tilde{\beta}(2+\tilde{\beta})(3+\tilde{\beta})\widetilde{R_T}^3+[3c^2(2+\tilde{\beta})(3+\tilde{\beta})+2c\tilde{\beta}(3+\tilde{\beta})+\tilde{\beta}^2]\widetilde{R_T}^2+[6c^3(3+\tilde{\beta})+c(3c+2)\tilde{\beta}]\widetilde{R_T}+6c^3\right]}{\widetilde{R_T}\left[(1+\tilde{\beta})(2+\tilde{\beta})(3+\tilde{\beta})\widetilde{R_T}^3+[3c^2(2+\tilde{\beta})(3+\tilde{\beta})+2c\tilde{\beta}(3+\tilde{\beta})+\tilde{\beta}(1+\tilde{\beta})]\widetilde{R_T}^2+c[6c^2(3+\tilde{\beta})+(3c+2)\tilde{\beta}]\widetilde{R_T}+6c^3\right]} \\ \frac{\tilde{\alpha}}{\tilde{\beta}}\frac{c^2\left[\tilde{\beta}(2+\tilde{\beta})(3+\tilde{\beta})\widetilde{R_T}^3+[3c^2(2+\tilde{\beta})(3+\tilde{\beta})+2c\tilde{\beta}(3+\tilde{\beta})+\tilde{\beta}^2]\widetilde{R_T}^2+[6c^3(3+\tilde{\beta})+c(3c+2)\tilde{\beta}]\widetilde{R_T}+6c^3\right]}{\widetilde{R_T}\left[(1+\tilde{\beta})(2+\tilde{\beta})(3+\tilde{\beta})\widetilde{R_T}^3+[3c^2(2+\tilde{\beta})(3+\tilde{\beta})+2c\tilde{\beta}(3+\tilde{\beta})+\tilde{\beta}(1+\tilde{\beta})]\widetilde{R_T}^2+c[6c^2(3+\tilde{\beta})+(3c+2)\tilde{\beta}]\widetilde{R_T}+6c^3\right]} \\ \frac{\tilde{\alpha}}{\tilde{\beta}}\frac{c^2\left[\tilde{\beta}(2+\tilde{\beta})(3+\tilde{\beta})\widetilde{R_T}^3+[3c^2(2+\tilde{\beta})(3+\tilde{\beta})+2c\tilde{\beta}(3+\tilde{\beta})+\tilde{\beta}^2]\widetilde{R_T}^2+[6c^3(3+\tilde{\beta})+c(3c+2)\tilde{\beta}]\widetilde{R_T}+6c^3\right]}{\widetilde{R_T}\left[(1+\tilde{\beta})(2+\tilde{\beta})(3+\tilde{\beta})\widetilde{R_T}^3+[3c^2(2+\tilde{\beta})(3+\tilde{\beta})+2c\tilde{\beta}(3+\tilde{\beta})+\tilde{\beta}(1+\tilde{\beta})]\widetilde{R_T}^2+c[6c^2(3+\tilde{\beta})+(3c+2)\tilde{\beta}]\widetilde{R_T}+6c^3\right]} \\ \frac{\tilde{\alpha}}{\tilde{\beta}}\frac{3c^2\left[(2+\tilde{\beta})(3+\tilde{\beta})\widetilde{R_T}^2+\left((2c+1)\tilde{\beta}+6c\right)\widetilde{R_T}+2c\right]}{(1+\tilde{\beta})(2+\tilde{\beta})(3+\tilde{\beta})\widetilde{R_T}^3+[3c^2(2+\tilde{\beta})(3+\tilde{\beta})+2c\tilde{\beta}(3+\tilde{\beta})+\tilde{\beta}(1+\tilde{\beta})]\widetilde{R_T}^2+c[6c^2(3+\tilde{\beta})+(3c+2)\tilde{\beta}]\widetilde{R_T}+6c^3} \end{bmatrix}, \tag{7}$$

As the total sum of components in m_1 and m_2 are the first moment $\langle m \rangle$ and second moment $\langle m^2 \rangle$ of the mRNA distribution, respectively, we can get the equations for $\langle m \rangle$ and $\langle m^2 \rangle$ by multiplying the vector $u = [1 \quad 1 \quad 1 \quad 1 \quad 1 \quad 1 \quad 1 \quad 1]$ Eq. (5) and Eq. (6) as follows:

$$0 = -\tilde{\beta}\langle m \rangle + u \cdot \tilde{P}m_0,$$

and

$$0 = -2\tilde{\beta}\langle m^2 \rangle + 2u \cdot \tilde{P}m_1 + \tilde{\beta}\langle m \rangle + u \cdot \tilde{P}m_0.$$

Therefore, we get the following equations:

$$\langle m \rangle = \frac{u \cdot \tilde{P}m_0}{\tilde{\beta}}, \tag{8}$$

and

$$\langle m^2 \rangle = \frac{u \cdot \tilde{P}m_1}{\tilde{\beta}} + \frac{\langle m \rangle}{2} + \frac{u \cdot \tilde{P}m_0}{2\tilde{\beta}} = \frac{u \cdot \tilde{P}m_1}{\tilde{\beta}} + \langle m \rangle. \tag{9}$$

Combining Eq. (4) and Eq. (7) with Eq. (8) and Eq. (9), we can get the following approximations for $\langle m \rangle$ and $\langle m^2 \rangle$ as follows:

$$\langle m\rangle \approx \frac{\tilde{\alpha}}{\tilde{\beta}}\left(1-\frac{\widetilde{R_T}^3}{c^3+3c^3\widetilde{R_T}+3c^2\widetilde{R_T}^2+\widetilde{R_T}^3}\right),$$

and

$$\langle m^2\rangle \approx \frac{\tilde{\alpha}}{\tilde{\beta}}\left(1-\frac{\widetilde{R_T}^3}{c^3+3c^3\widetilde{R_T}+3c^2\widetilde{R_T}^2+\widetilde{R_T}^3}\right)\times$$

$$\left(1+\frac{\tilde{\alpha}}{\tilde{\beta}}\frac{3\tilde{\beta}(2+\tilde{\beta})(3+\tilde{\beta})\widetilde{R_T}^5+3\left(18c^2+15c(1+c)\tilde{\beta}+(1+8c+3c^2)\tilde{\beta}^2+c\tilde{\beta}^3\right)\widetilde{R_T}^4+c\left(108c^2+(20+27c+63c^2)\tilde{\beta}+3(3+2c+3c^2)\tilde{\beta}^2+\tilde{\beta}^3\right)\widetilde{R_T}^3+c\left(18(2+3c)c^2+(1+12c+24c^2+18c^3)\tilde{\beta}+(1+2c+3c^2)\tilde{\beta}^2\right)\widetilde{R_T}^2+c^2\left(36c^2+(2+3c+6c^2)\tilde{\beta}\right)\widetilde{R_T}+6c^4}{\left(3\widetilde{R_T}^2+3c\widetilde{R_T}+c\right)\left[(6+11\tilde{\beta}+6\tilde{\beta}^2+\tilde{\beta}^3)\widetilde{R_T}^3+\left(18c^2+(1+6c+15c^2)\tilde{\beta}+(1+2c+3c^2)\tilde{\beta}^2\right)\widetilde{R_T}^2+c\left(18c^2+(2+3c+6c^2)\tilde{\beta}\right)\widetilde{R_T}+6c^3\right]}\right).$$

Using these, we can obtain the Fano factor of m, $FF(\widetilde{R_T})$, defined as $\frac{\langle m^2\rangle-\langle m\rangle^2}{\langle m\rangle}$ (Supplementary Table 2) as follows:

$$FF(\widetilde{R_T}) = 1+\frac{\tilde{\alpha}\widetilde{R_T}^4\left[3(\tilde{\beta}+2)(\tilde{\beta}+3)\widetilde{R_T}^4+\left[3c\tilde{\beta}^2+(24c+3)\tilde{\beta}+45c\right]\widetilde{R_T}^3+\left[c\tilde{\beta}^2+3c(2c+3)\tilde{\beta}+2c(9c+10)\right]\widetilde{R_T}^2+c\left[(1+2c)\tilde{\beta}+(1+12c)\right]\widetilde{R_T}+2c^2\right]}{\begin{array}{r}3(\tilde{\beta}+1)(\tilde{\beta}+2)(\tilde{\beta}+3)\widetilde{R_T}^8+3\left[c(3c+1)\tilde{\beta}^3+(21c^2+8c+1)\tilde{\beta}^2+(48c^2+17c+1)\tilde{\beta}+6c(1+6c)\right]\widetilde{R_T}^7 \\ +c\left[6(1+36c^2+27c^3)+(20+36c+315c^2+135c^3)\tilde{\beta}+3(3+5c+45c^2+9c^3)\tilde{\beta}^2+(1+18c^2)\tilde{\beta}^3\right]\widetilde{R_T}^6 \\ +c\left[3c^2(3c+2)\tilde{\beta}^3+(54c^4+90c^3+57c^2+2c+1)\tilde{\beta}^2+(324c^4+252c^3+126c^2+12c+1)\tilde{\beta}+18c^2(27c^2+6c+4)\right]\widetilde{R_T}^5 \\ +c^2\left[6c^2\tilde{\beta}^3+3c(9c^3+12c^2+19c+2)\tilde{\beta}^2+(243c^4+198c^3+153c^2+9c+2)\tilde{\beta}+18c^2(27c^2+9c+4)\right]\widetilde{R_T}^4 \\ +c^4\left[\tilde{\beta}^3+6(3c^2+2c+2)\tilde{\beta}^2+(54c^3+153c^2+72c+29)\tilde{\beta}+6(27c^3+54c^2+2)\right]\widetilde{R_T}^3 \\ +c^4\left[(3c^2+2c+1)\tilde{\beta}^2+(36c^3+33c^2+18c+1)\tilde{\beta}+54c^2(1+3c)\right]\widetilde{R_T}^2+c^5\left[(6c^2+3c+2)\tilde{\beta}+54c^2\right]\widetilde{R_T}+6c^7\end{array}}.$$

Supplementary Note 2. The equations for the transcriptional activity and the Fano factor of the model describing the sequestration

The transcription regulated by the sole sequestration (Fig. 2a and Supplementary Table 1) can be described using Eq. (1) with

$$K=\begin{bmatrix}-k_fA & k_a\\ k_fA & -k_a\end{bmatrix},\ P=\begin{bmatrix}0 & 0\\ 0 & \alpha\end{bmatrix},\text{ and } p_m=\begin{bmatrix}p_m^F\\ p_m^A\end{bmatrix},$$

where p_m^X is the joint probability that DNA is in state E_X with m mRNAs, $X\in\{F,A\}$, k_f and k_a are the association and dissociation rates between free activator A and DNA, respectively. To calculate the stationary mean and variance of mRNAs in this system, we first derived the stationary mean of A, $\langle A\rangle$, in the matrix K. Specifically, $\langle A\rangle$ can be derived from the CME describing the reversible binding (i.e., sequestration) between A and free repressor R,

$$\langle RA\rangle = K_s\langle R_A\rangle, \qquad (10)$$

where K_s is the dissociation constant between A and R.

As the total numbers of the activator ($A_T=A+R_A+E_A$) and the repressor ($R_T=R+R_A$) are conserved and the total number of the activator is much larger than that of DNA (i.e., $A_T\gg 1$),

R_A and R in Eq. (10) can be replaced with $A_T - A - E_A \approx A_T - A$ and $R_T - (A_T - A - E_A) \approx R_T - (A_T - A)$. Consequently, we can get the equation for the first and the second moments of A as follows:

$$\langle A^2\rangle - (A_T - R_T - K_s)\langle A\rangle - K_s A_T = 0. \quad (11)$$

Assuming that $\langle A^2\rangle \approx \langle A\rangle^2$ (i.e., $Var(A) = \langle A^2\rangle - \langle A\rangle^2 \approx 0$), Eq. (11) can be switched to the quadratic equation for $\langle A\rangle$. Then, we can get the approximated equation for $\langle A\rangle$, and thus the approximated equations for $\langle R\rangle$ and $\langle R_A\rangle$ as follows:

$$\begin{aligned}
\langle A\rangle &\approx \frac{A_T - R_T - K_s + \sqrt{(A_T - B_T - K_s)^2 + 4A_T K_s}}{2} \coloneqq A(A_T, R_T, K_s),\\
\langle R\rangle &\approx R_T - \big(A_T - A(A_T, R_T, K_s)\big) \coloneqq R(A_T, R_T, K_s), \qquad (12)\\
\langle R_A\rangle &\approx R_T - R(A_T, R_T, K_s) \coloneqq R_A(A_T, R_T, K_s).
\end{aligned}$$

These approximations are accurate as long as the number of the activator is sufficiently large $(A_T > 10K_s^{-1})$[2-5]. Please see Song *et al.* (2021) for a detailed description to derive this condition.

Next, substituting Eq. (12) for A in the matrix K and rescaling time variable t by $k_f A_T$ to reduce the number of parameters, we can rewrite the CME describing the sole sequestration as Eq. (2), where

$$\widetilde{K} = \begin{bmatrix} -\tilde{A}(\widetilde{R_T}, \widetilde{K_s}) & \widetilde{K_a} \\ \tilde{A}(\widetilde{R_T}, \widetilde{K_s}) & -\widetilde{K_a} \end{bmatrix},\ \tilde{P} = \begin{bmatrix} 0 & 0 \\ 0 & \tilde{\alpha} \end{bmatrix}, \text{ and } p_m = \begin{bmatrix} p_m^F \\ p_m^A \end{bmatrix}.$$

Here, $K_a = k_a/k_f$ is the dissociation constant between free activator A and DNA, $\tilde{X} = X/A_T$, $X \in \{A, R_T, K_s, K_a\}$, $\tilde{\alpha} = \alpha/k_f A_T$, and $\tilde{\beta} = \beta/k_f A_T$. Then, by solving the steady state equation $0 = \widetilde{K} m_0$ and Eq. (5), we can get

$$m_0 \approx \begin{bmatrix} \dfrac{1}{1+\frac{\tilde{A}(\widetilde{R_T},\widetilde{K_s})}{\widetilde{K_a}}} \\ \dfrac{\frac{\tilde{A}(\widetilde{R_T},\widetilde{K_s})}{\widetilde{K_a}}}{1+\frac{\tilde{A}(\widetilde{R_T},\widetilde{K_s})}{\widetilde{K_a}}} \end{bmatrix} \text{ and } m_1 \approx \begin{bmatrix} \dfrac{\tilde{\alpha}}{\tilde{\beta}} \dfrac{\tilde{A}(\widetilde{R_T},\widetilde{K_s})}{\left(1+\frac{\tilde{A}(\widetilde{R_T},\widetilde{K_s})}{\widetilde{K_a}}\right)\left(\tilde{A}(\widetilde{R_T},\widetilde{K_s})+\widetilde{K_a}+\tilde{\beta}\right)} \\ \dfrac{\tilde{\alpha}}{\tilde{\beta}} \dfrac{\frac{\tilde{A}(\widetilde{R_T},\widetilde{K_s})}{\widetilde{K_a}}\left(\tilde{A}(\widetilde{R_T},\widetilde{K_s})+\tilde{\beta}\right)}{\left(1+\frac{\tilde{A}(\widetilde{R_T},\widetilde{K_s})}{\widetilde{K_a}}\right)\left(\tilde{A}(\widetilde{R_T},\widetilde{K_s})+\widetilde{K_a}+\tilde{\beta}\right)} \end{bmatrix}. \quad (13)$$

The transcriptional activity, $TA(\widetilde{R_T})$, is defined as the probability that DNA is activated, and thus it is the second element of m_0 (Supplementary Table 2):

$$TA(\widetilde{R_T}) = \frac{\tilde{A}(\widetilde{R_T},\widetilde{K_s})/\widetilde{K_a}}{1+\tilde{A}(\widetilde{R_T},\widetilde{K_s})/\widetilde{K_a}}.$$

Subsequently, substituting m_0 and m_1 to Eq. (8) and Eq. (9), we can get the following approximations for $\langle m \rangle$ and $\langle m^2 \rangle$ as follows:

$$\langle m \rangle \approx \frac{\tilde{\alpha}}{\tilde{\beta}} \frac{\frac{\tilde{A}(\widetilde{R_T},\widetilde{K_s})}{\widetilde{K_a}}}{1+\frac{\tilde{A}(\widetilde{R_T},\widetilde{K_s})}{\widetilde{K_a}}}, \tag{14}$$

and

$$\langle m^2 \rangle \approx \frac{\tilde{\alpha}}{\tilde{\beta}} \frac{\frac{\tilde{A}(\widetilde{R_T},\widetilde{K_s})}{\widetilde{K_a}}}{1+\frac{\tilde{A}(\widetilde{R_T},\widetilde{K_s})}{\widetilde{K_a}}} \left(1+\frac{\tilde{\alpha}}{\tilde{\beta}} \frac{\tilde{A}(\widetilde{R_T},\widetilde{K_s})+\tilde{\beta}}{\tilde{A}(\widetilde{R_T},\widetilde{K_s})+\widetilde{K_a}+\tilde{\beta}}\right). \tag{15}$$

Using these, we can obtain the Fano factor of m, $FF(\widetilde{R_T})$, defined as $\frac{\langle m^2 \rangle - \langle m \rangle^2}{\langle m \rangle}$ (Supplementary Table 2) as follows:

$$FF(\widetilde{R_T}) = 1 + \frac{\tilde{\alpha}}{(1+\tilde{A}(\widetilde{R_T},\widetilde{K_s})/\widetilde{K_a})(\tilde{A}(\widetilde{R_T},\widetilde{K_s})+\widetilde{K_a}+\tilde{\beta})}.$$

Supplementary Note 3. The equations for the transcriptional activity and the Fano factor of the model describing the sequestration and blocking

The transcription regulated by the combination of the sequestration and blocking (Fig. 3a and Supplementary Table 1) can be described using Eq. (1) with

$$K = \begin{bmatrix} -k_f A & k_a & 0 \\ k_f A & -k_a - k_f R & k_b \\ 0 & k_f R & -k_b \end{bmatrix}, \; P = \begin{bmatrix} 0 & 0 & 0 \\ 0 & \alpha & 0 \\ 0 & 0 & 0 \end{bmatrix}, \text{ and } p_m = \begin{bmatrix} p_m^F \\ p_m^A \\ p_m^R \end{bmatrix},$$

where p_m^X is the joint probability that DNA is in state E_X with m mRNAs, $X \in \{F, A, R\}$, k_f and k_b are the association and dissociation rates between free repressor R and the DNA-bound activator, respectively. As done in the model describing the sole sequestration, we derived the stationary means of A, R, and R_A as in Eq. (12). Then, rescaling time variable t by $k_f A_T$, we can rewrite the CME describing the sequestration and blocking as Eq. (2), where

$$\widetilde{K} = \begin{bmatrix} -\tilde{A}\left(\widetilde{R_T}, \widetilde{K_s}\right) & \widetilde{K_a} & 0 \\ \tilde{A}\left(\widetilde{R_T}, \widetilde{K_s}\right) & -\widetilde{K_a} - \tilde{R}\left(\widetilde{R_T}, \widetilde{K_s}\right) & \widetilde{K_b} \\ 0 & \tilde{R}\left(\widetilde{R_T}, \widetilde{K_s}\right) & -\widetilde{K_b} \end{bmatrix}, \ \tilde{P} = \begin{bmatrix} 0 & 0 & 0 \\ 0 & \tilde{\alpha} & 0 \\ 0 & 0 & 0 \end{bmatrix}, \text{ and } p_m = \begin{bmatrix} p_m^F \\ p_m^A \\ p_m^R \end{bmatrix}.$$

Here, $K_b = k_b/k_f$ is the dissociation constant between free activator R and the DNA-bound activator, $\tilde{X} = X/A_T$, $X \in \{A, R, R_T, K_s, K_a, K_b\}$, $\tilde{\alpha} = \alpha/k_f A_T$, and $\tilde{\beta} = \beta/k_f A_T$. Then, by solving the steady state equation $0 = \widetilde{K} m_0$, we can get

$$m_0 \approx \begin{bmatrix} \dfrac{1}{1 + \dfrac{\tilde{A}\left(\widetilde{R_T}, \widetilde{K_s}\right)}{\widetilde{K_a}} + \dfrac{\tilde{A}\left(\widetilde{R_T}, \widetilde{K_s}\right)}{\widetilde{K_a}} \dfrac{\tilde{R}\left(\widetilde{R_T}, \widetilde{K_s}\right)}{\widetilde{K_b}}} \\ \dfrac{\dfrac{\tilde{A}\left(\widetilde{R_T}, \widetilde{K_s}\right)}{\widetilde{K_a}}}{1 + \dfrac{\tilde{A}\left(\widetilde{R_T}, \widetilde{K_s}\right)}{\widetilde{K_a}} + \dfrac{\tilde{A}\left(\widetilde{R_T}, \widetilde{K_s}\right)}{\widetilde{K_a}} \dfrac{\tilde{R}\left(\widetilde{R_T}, \widetilde{K_s}\right)}{\widetilde{K_b}}} \\ \dfrac{\dfrac{\tilde{A}\left(\widetilde{R_T}, \widetilde{K_s}\right)}{\widetilde{K_a}} \dfrac{\tilde{R}\left(\widetilde{R_T}, \widetilde{K_s}\right)}{\widetilde{K_b}}}{1 + \dfrac{\tilde{A}\left(\widetilde{R_T}, \widetilde{K_s}\right)}{\widetilde{K_a}} + \dfrac{\tilde{A}\left(\widetilde{R_T}, \widetilde{K_s}\right)}{\widetilde{K_a}} \dfrac{\tilde{R}\left(\widetilde{R_T}, \widetilde{K_s}\right)}{\widetilde{K_b}}} \end{bmatrix}$$

The transcriptional activity, $TA\left(\widetilde{R_T}\right)$, is defined as the probability that DNA is activated, and thus it is the second element of m_0 (Supplementary Table 2):

$$TA\left(\widetilde{R_T}\right) = \frac{\dfrac{\tilde{A}\left(\widetilde{R_T}, \widetilde{K_s}\right)}{\widetilde{K_a}}}{1 + \dfrac{\tilde{A}\left(\widetilde{R_T}, \widetilde{K_s}\right)}{\widetilde{K_a}} + \dfrac{\tilde{A}\left(\widetilde{R_T}, \widetilde{K_s}\right)}{\widetilde{K_a}} \dfrac{\tilde{R}\left(\widetilde{R_T}, \widetilde{K_s}\right)}{\widetilde{K_b}}}.$$

Similarly, by solving Eq. (5), we can get

$$m_1 \approx \begin{bmatrix} \frac{\tilde{\alpha}}{\tilde{\beta}} \frac{\frac{\tilde{A}(\widetilde{R_T}, \widetilde{K_s})}{\widetilde{K_a}}}{1 + \frac{\tilde{A}(\widetilde{R_T}, \widetilde{K_s})}{\widetilde{K_a}} + \frac{\tilde{A}(\widetilde{R_T}, \widetilde{K_s})}{\widetilde{K_a}} \frac{\tilde{R}(\widetilde{R_T}, \widetilde{K_s})}{\widetilde{K_b}}} \frac{\widetilde{K_a}(\widetilde{K_b} + \tilde{\beta})}{[\widetilde{K_a}(\widetilde{K_b} + \tilde{\beta}) + (\tilde{A}(\widetilde{R_T}, \widetilde{K_s}) + \tilde{\beta})(\widetilde{K_b} + \tilde{\beta}) + (\tilde{A}(\widetilde{R_T}, \widetilde{K_s}) + \tilde{\beta})\tilde{R}(\widetilde{R_T}, \widetilde{K_s})]} \\ \frac{\tilde{\alpha}}{\tilde{\beta}} \frac{\frac{\tilde{A}(\widetilde{R_T}, \widetilde{K_s})}{\widetilde{K_a}}}{1 + \frac{\tilde{A}(\widetilde{R_T}, \widetilde{K_s})}{\widetilde{K_a}} + \frac{\tilde{A}(\widetilde{R_T}, \widetilde{K_s})}{\widetilde{K_a}} \frac{\tilde{R}(\widetilde{R_T}, \widetilde{K_s})}{\widetilde{K_b}}} \frac{(\tilde{A}(\widetilde{R_T}, \widetilde{K_s}) + \tilde{\beta})(\widetilde{K_b} + \tilde{\beta})}{[\widetilde{K_a}(\widetilde{K_b} + \tilde{\beta}) + (\tilde{A}(\widetilde{R_T}, \widetilde{K_s}) + \tilde{\beta})(\widetilde{K_b} + \tilde{\beta}) + (\tilde{A}(\widetilde{R_T}, \widetilde{K_s}) + \tilde{\beta})\tilde{R}(\widetilde{R_T}, \widetilde{K_{\text{[illegible]}}})]} \\ \frac{\tilde{\alpha}}{\tilde{\beta}} \frac{\frac{\tilde{A}(\widetilde{R_T}, \widetilde{K_s})}{\widetilde{K_a}}}{1 + \frac{\tilde{A}(\widetilde{R_T}, \widetilde{K_s})}{\widetilde{K_a}} + \frac{\tilde{A}(\widetilde{R_T}, \widetilde{K_s})}{\widetilde{K_a}} \frac{\tilde{R}(\widetilde{R_T}, \widetilde{K_s})}{\widetilde{K_b}}} \frac{(\tilde{A}(\widetilde{R_T}, \widetilde{K_s}) + \tilde{\beta})\tilde{R}(\widetilde{R_T}, \widetilde{K_s})}{[\widetilde{K_a}(\widetilde{K_b} + \tilde{\beta}) + (\tilde{A}(\widetilde{R_T}, \widetilde{K_s}) + \tilde{\beta})(\widetilde{K_b} + \tilde{\beta}) + (\tilde{A}(\widetilde{R_T}, \widetilde{K_s}) + \tilde{\beta})\tilde{R}(\widetilde{R_T}, \widetilde{K_s})]} \end{bmatrix}.$$

Subsequently, substituting m_0 and m_1 to Eq. (8) and Eq. (9), we can get the following approximations for $\langle m \rangle$ and $\langle m^2 \rangle$ as follows:

$$\langle m \rangle \approx \frac{\tilde{\alpha}}{\tilde{\beta}} \frac{\frac{\tilde{A}(\widetilde{R_T}, \widetilde{K_s})}{\widetilde{K_a}}}{1 + \frac{\tilde{A}(\widetilde{R_T}, \widetilde{K_s})}{\widetilde{K_a}} + \frac{\tilde{A}(\widetilde{R_T}, \widetilde{K_s})}{\widetilde{K_a}} \frac{\tilde{R}(\widetilde{R_T}, \widetilde{K_s})}{\widetilde{K_b}}}, \tag{16}$$

and

$$\langle m^2 \rangle \approx \frac{\tilde{\alpha}}{\tilde{\beta}} \frac{\frac{\tilde{A}(\widetilde{R_T}, \widetilde{K_s})}{\widetilde{K_a}}}{1 + \frac{\tilde{A}(\widetilde{R_T}, \widetilde{K_s})}{\widetilde{K_a}} + \frac{\tilde{A}(\widetilde{R_T}, \widetilde{K_s})}{\widetilde{K_a}} \frac{\tilde{R}(\widetilde{R_T}, \widetilde{K_s})}{\widetilde{K_b}}} \times$$
$$\left(1 + \frac{\tilde{\alpha}}{\tilde{\beta}} \frac{\left(\tilde{A}(\widetilde{R_T}, \widetilde{K_s}) + \tilde{\beta}\right)\left(\widetilde{K_b} + \tilde{\beta}\right)}{\left[\widetilde{K_a}\left(\widetilde{K_b} + \tilde{\beta}\right) + \left(\tilde{A}(\widetilde{R_T}, \widetilde{K_s}) + \tilde{\beta}\right)\left(\widetilde{K_b} + \tilde{\beta}\right) + \left(\tilde{A}(\widetilde{R_T}, \widetilde{K_s}) + \tilde{\beta}\right)\tilde{R}(\widetilde{R_T}, \widetilde{K_s})\right]}\right). \tag{17}$$

Using these, we can obtain the Fano factor of m, $FF(\widetilde{R_T})$, defined as $\frac{\langle m^2 \rangle - \langle m \rangle^2}{\langle m \rangle}$ (Supplementary Table 2) as follows:

$$FF(\widetilde{R_T}) = 1 + \frac{\tilde{\alpha}\left[\widetilde{K_b} + \tilde{\beta} + \left(\tilde{A}(\widetilde{R_T}, \widetilde{K_s}) + \tilde{\beta}\right)\frac{\tilde{A}(\widetilde{R_T}, \widetilde{K_s})}{\widetilde{K_a}}\frac{\tilde{R}(\widetilde{R_T}, \widetilde{K_s})}{\widetilde{K_b}}\right]}{\left(1 + \frac{\tilde{A}(\widetilde{R_T}, \widetilde{K_s})}{\widetilde{K_a}} + \frac{\tilde{A}(\widetilde{R_T}, \widetilde{K_s})}{\widetilde{K_a}}\frac{\tilde{R}(\widetilde{R_T}, \widetilde{K_s})}{\widetilde{K_b}}\right)\left[\widetilde{K_a}\left(\widetilde{K_b} + \tilde{\beta}\right) + \left(\tilde{A}(\widetilde{R_T}, \widetilde{K_s}) + \tilde{\beta}\right)\left(\tilde{R}(\widetilde{R_T}, \widetilde{K_s}) + \widetilde{K_b} + \tilde{\beta}\right)\right]}.$$

Supplementary Note 4. The equations for the transcriptional activity and the Fano factor of the model describing the sequestration, blocking, and displacement

The transcription regulated by the combination of the sequestration, blocking, and displacement (Fig. 4a and Supplementary Table 1) can be described using Eq. (1) with

$$K = \begin{bmatrix} -k_f A - k_f R_A & k_a & k_d \\ k_f A & -k_a - k_f R & k_b \\ k_f R_A & k_f R & -k_b - k_d \end{bmatrix}, \ P = \begin{bmatrix} 0 & 0 & 0 \\ 0 & \alpha & 0 \\ 0 & 0 & 0 \end{bmatrix}, \text{ and } p_m = \begin{bmatrix} p_m^F \\ p_m^A \\ p_m^R \end{bmatrix},$$

where p_m^X is the joint probability that DNA is in state E_X with m mRNAs, $X \in \{F, A, R\}$, k_f and k_d are the association and dissociation rates between the repressor-activator complex R_A and DNA, respectively. As done in models describing the sole sequestration and describing the sequestration and blocking, we derived the stationary means of A, R, and R_A as in Eq. (12). Then, rescaling time variable t by $k_f A_T$, we can rewrite the CME describing the sequestration, blocking, and displacement as Eq. (2), where

$$\tilde{K}=\begin{bmatrix}-\tilde{A}(\widetilde{R_T},\widetilde{K_s})-\widetilde{R_A}(\widetilde{R_T},\widetilde{K_s}) & \widetilde{K_a} & \widetilde{K_d}\\ \tilde{A}(\widetilde{R_T},\widetilde{K_s}) & -\widetilde{K_a}-\tilde{R}(\widetilde{R_T},\widetilde{K_s}) & \widetilde{K_b}\\ \widetilde{R_A}(\widetilde{R_T},\widetilde{K_s}) & \tilde{R}(\widetilde{R_T},\widetilde{K_s}) & -\widetilde{K_b}-\widetilde{K_d}\end{bmatrix},\ \tilde{P}=\begin{bmatrix}0&0&0\\0&\tilde{\alpha}&0\\0&0&0\end{bmatrix},\text{ and } p_m=\begin{bmatrix}p_m^F\\p_m^A\\p_m^R\end{bmatrix}.$$

Here, $K_d=k_b/k_f$ is the dissociation constant between the repressor-activator complex R_A and DNA, $\tilde{X}=X/A_T$, $X\in\{A,R,R_A,R_T,K_s,K_a,K_b,K_d\}$, $\tilde{\alpha}=\alpha/k_fA_T$, and $\tilde{\beta}=\beta/k_fA_T$. Then, by solving the steady state equation $0=\tilde{K}m_0$, we can get

$$m_0\approx\begin{bmatrix}\dfrac{1}{1+\tilde{I}(\widetilde{R_T},\widetilde{K_a},\widetilde{K_s})\dfrac{\tilde{A}(\widetilde{R_T},\widetilde{K_s})}{\widetilde{K_a}}+\tilde{J}(\widetilde{R_T},\widetilde{K_a},\widetilde{K_s})\dfrac{\tilde{A}(\widetilde{R_T},\widetilde{K_s})}{\widetilde{K_a}}\dfrac{\tilde{R}(\widetilde{R_T},\widetilde{K_s})}{\widetilde{K_b}}}\\ \dfrac{\tilde{I}(\widetilde{R_T},\widetilde{K_a},\widetilde{K_s})\dfrac{\tilde{A}(\widetilde{R_T},\widetilde{K_s})}{\widetilde{K_a}}}{1+\tilde{I}(\widetilde{R_T},\widetilde{K_a},\widetilde{K_s})\dfrac{\tilde{A}(\widetilde{R_T},\widetilde{K_s})}{\widetilde{K_a}}+\tilde{J}(\widetilde{R_T},\widetilde{K_a},\widetilde{K_s})\dfrac{\tilde{A}(\widetilde{R_T},\widetilde{K_s})}{\widetilde{K_a}}\dfrac{\tilde{R}(\widetilde{R_T},\widetilde{K_s})}{\widetilde{K_b}}}\\ \dfrac{\tilde{J}(\widetilde{R_T},\widetilde{K_a},\widetilde{K_s})\dfrac{\tilde{A}(\widetilde{R_T},\widetilde{K_s})}{\widetilde{K_a}}\dfrac{\tilde{R}(\widetilde{R_T},\widetilde{K_s})}{\widetilde{K_b}}}{1+\tilde{I}(\widetilde{R_T},\widetilde{K_a},\widetilde{K_s})\dfrac{\tilde{A}(\widetilde{R_T},\widetilde{K_s})}{\widetilde{K_a}}+\tilde{J}(\widetilde{R_T},\widetilde{K_a},\text{⍰⍰}_s)\dfrac{\tilde{A}(\widetilde{R_T},\widetilde{K_s})}{\widetilde{K_a}}\dfrac{\tilde{R}(\widetilde{R_T},\widetilde{K_s})}{\widetilde{K_b}}}\end{bmatrix}.$$

The transcriptional activity, $TA(\widetilde{R_T})$, is defined as the probability that DNA is activated, and thus it is the second element of m_0 (Supplementary Table 2):

$$TA(\widetilde{R_T})=\frac{\tilde{I}(\widetilde{R_T},\widetilde{K_a},\widetilde{K_s})\dfrac{\tilde{A}(\widetilde{R_T},\widetilde{K_s})}{\widetilde{K_a}}}{1+\tilde{I}(\widetilde{R_T},\widetilde{K_a},\widetilde{K_s})\dfrac{\tilde{A}(\widetilde{R_T},\widetilde{K_s})}{\widetilde{K_a}}+\tilde{J}(\widetilde{R_T},\widetilde{K_a},\widetilde{K_s})\dfrac{\tilde{A}(\widetilde{R_T},\widetilde{K_s})}{\widetilde{K_a}}\dfrac{\tilde{R}(\widetilde{R_T},\widetilde{K_s})}{\widetilde{K_b}}}.$$

Similarly, by solving Eq. (5), we can get

$$m_1\approx\begin{bmatrix}\frac{\tilde{\alpha}}{\tilde{\beta}}\frac{\tilde{I}(\widetilde{R_T},\widetilde{K_a},\widetilde{K_s})\frac{\tilde{A}(\widetilde{R_T},\widetilde{K_s})}{\widetilde{K_a}}}{1+\tilde{I}(\widetilde{R_T},\widetilde{K_a},\widetilde{K_s})\frac{\tilde{A}(\widetilde{R_T},\widetilde{K_s})}{\widetilde{K_a}}+\tilde{J}(\widetilde{R_T},\widetilde{K_a},\widetilde{K_s})\frac{\tilde{A}(\widetilde{R_T},\widetilde{K_s})}{\widetilde{K_a}}\frac{\tilde{R}(\widetilde{R_T},\widetilde{K_s})}{\widetilde{K_b}}}\frac{(\widetilde{K_b}+\widetilde{K_d}+\tilde{\beta})\widetilde{K_a}+\widetilde{K_d}\tilde{R}(\widetilde{R_T},\widetilde{K_s})}{[(\widetilde{K_b}+\widetilde{K_d}+\tilde{\beta})(\tilde{A}(\widetilde{R_T},\widetilde{K_s})+\widetilde{K_a}+\tilde{\beta})+(\widetilde{K_b}+\widetilde{K_a}+\tilde{\beta})\widetilde{R_A}(\widetilde{R_T},\widetilde{K_s})+(1+\widetilde{K_d}+\tilde{\beta})\tilde{R}(\widetilde{R_T},\widetilde{K_s})]}\\ \frac{\tilde{\alpha}}{\tilde{\beta}}\frac{\tilde{I}(\widetilde{R_T},\widetilde{K_a},\widetilde{K_s})\frac{\tilde{A}(\widetilde{R_T},\widetilde{K_s})}{\widetilde{K_a}}}{1+\tilde{I}(\widetilde{R_T},\widetilde{K_a},\widetilde{K_s})\frac{\tilde{A}(\widetilde{R_T},\widetilde{K_s})}{\widetilde{K_a}}+\tilde{J}(\widetilde{R_T},\widetilde{K_a},\widetilde{K_s})\frac{\tilde{A}(\widetilde{R_T},\widetilde{K_s})}{\widetilde{K_a}}\frac{\tilde{R}(\widetilde{R_T},\widetilde{K_s})}{\widetilde{K_b}}}\frac{(\widetilde{K_b}+\widetilde{K_d}+\tilde{\beta})(\tilde{A}(\widetilde{R_T},\widetilde{K_s})+\tilde{\beta})+\widetilde{R_A}(\widetilde{R_T},\widetilde{K_s})(\widetilde{K_b}+\tilde{\beta})}{[(\widetilde{K_b}+\widetilde{K_d}+\tilde{\beta})(\tilde{A}(\widetilde{R_T},\widetilde{K_s})+\widetilde{K_a}+\tilde{\beta})+(\widetilde{K_b}+\widetilde{K_a}+\tilde{\beta})\widetilde{R_A}(\widetilde{R_T},\widetilde{K_s})+(1+\widetilde{K_d}+\tilde{\beta})\tilde{R}(\widetilde{R_T},\widetilde{K_s})]}\\ \frac{\tilde{\alpha}}{\tilde{\beta}}\frac{\tilde{I}(\widetilde{R_T},\widetilde{K_a},\widetilde{K_s})\frac{\tilde{A}(\widetilde{R_T},\widetilde{K_s})}{\widetilde{K_a}}}{1+\tilde{I}(\widetilde{R_T},\widetilde{K_a},\widetilde{K_s})\frac{\tilde{A}(\widetilde{R_T},\widetilde{K_s})}{\widetilde{K_a}}+\tilde{J}(\widetilde{R_T},\widetilde{K_a},\widetilde{K_s})\frac{\tilde{A}(\widetilde{R_T},\widetilde{K_s})}{\widetilde{K_a}}\frac{\tilde{R}(\widetilde{R_T},\widetilde{K_s})}{\widetilde{K_b}}}\frac{(1+\tilde{\beta})\tilde{R}(\widetilde{R_T},\widetilde{K_s})+\widetilde{K_a}\widetilde{R_A}(\widetilde{R_T},\widetilde{K_s})}{[(\widetilde{K_b}+\widetilde{K_d}+\tilde{\beta})(\tilde{A}(\widetilde{R_T},\widetilde{K_s})+\widetilde{K_a}+\tilde{\beta})+(\widetilde{K_b}+\widetilde{K_a}+\tilde{\beta})\widetilde{R_A}(\widetilde{R_T},\widetilde{K_s})+(1+\widetilde{K_d}+\tilde{\beta})\tilde{R}(\widetilde{R_T},\widetilde{K_s})]}\end{bmatrix},$$

where $\tilde{I}(\widetilde{R_T},\widetilde{K_a},\widetilde{K_s})=\frac{\widetilde{K_s}+\sigma\widetilde{K_a}+\tilde{R}(\widetilde{R_T},\widetilde{K_s})}{\widetilde{K_s}+\sigma\widetilde{K_a}+\sigma\tilde{R}(\widetilde{R_T},\widetilde{K_s})}$, $\tilde{J}(\widetilde{R_T},\widetilde{K_a},\widetilde{K_s})=\frac{\widetilde{K_s}+\sigma\widetilde{K_a}+\sigma\tilde{R}(\widetilde{R_T},\widetilde{K_s})}{\widetilde{K_s}+\sigma\widetilde{K_a}+\sigma\tilde{R}(\widetilde{R_T},\widetilde{K_s})}$, and $\sigma=\frac{\widetilde{K_s}\widetilde{K_d}}{\widetilde{K_a}\widetilde{K_b}}$.

Subsequently, substituting m_0 and m_1 to Eq. (8) and Eq. (9), we can get the following approximations for $\langle m\rangle$ and $\langle m^2\rangle$ as follows:

$$\langle m\rangle \approx \frac{\tilde{\alpha}}{\tilde{\beta}} \frac{\tilde{I}(\widetilde{R_T},\widetilde{K_a},\widetilde{K_s})\frac{\tilde{A}(\widetilde{R_T},\widetilde{K_s})}{\widetilde{K_a}}}{1+\tilde{I}(\widetilde{R_T},\widetilde{K_a},\widetilde{K_s})\frac{\tilde{A}(\widetilde{R_T},\widetilde{K_s})}{\widetilde{K_a}}+\tilde{J}(\widetilde{R_T},\widetilde{K_a},\widetilde{K_s})\frac{\tilde{A}(\widetilde{R_T},\widetilde{K_s})}{\widetilde{K_a}}\frac{\tilde{R}(\widetilde{R_T},\widetilde{K_s})}{\widetilde{K_b}}}, \tag{18}$$

and

$$\langle m^2\rangle \approx \frac{\tilde{\alpha}}{\tilde{\beta}} \frac{\tilde{I}\left(\widetilde{R_T},\widetilde{K_a},\widetilde{K_s}\right)\frac{\tilde{A}\left(\widetilde{R_T},\widetilde{K_s}\right)}{\widetilde{K_a}}}{1+\tilde{I}\left(\widetilde{R_T},\widetilde{K_a},\widetilde{K_s}\right)\frac{\tilde{A}\left(\widetilde{R_T},\widetilde{K_s}\right)}{\widetilde{K_a}}+\tilde{J}\left(\widetilde{R_T},\widetilde{K_a},\widetilde{K_s}\right)\frac{\tilde{A}\left(\widetilde{R_T},\widetilde{K_s}\right)}{\widetilde{K_a}}\frac{\tilde{R}\left(\widetilde{R_T},\widetilde{K_s}\right)}{\widetilde{K_b}}} \times \\ \left(1+\frac{\tilde{\alpha}}{\tilde{\beta}} \frac{\left(\widetilde{K_b}+\widetilde{K_d}+\tilde{\beta}\right)\left(\tilde{A}\left(\widetilde{R_T},\widetilde{K_s}\right)+\tilde{\beta}\right)+\widetilde{R_A}\left(\widetilde{R_T},\widetilde{K_s}\right)\left(\widetilde{K_b}+\tilde{\beta}\right)}{\left[\left(\widetilde{K_b}+\widetilde{K_d}+\tilde{\beta}\right)\left(\tilde{A}\left(\widetilde{R_T},\widetilde{K_s}\right)+\widetilde{K_a}+\tilde{\beta}\right)+\left(\widetilde{K_b}+\widetilde{K_a}+\tilde{\beta}\right)\widetilde{R_A}\left(\widetilde{R_T},\widetilde{K_s}\right)+\left(1+\widetilde{K_d}+\tilde{\beta}\right)\tilde{R}\left(\widetilde{R_T},\widetilde{K_s}\right)\right]}\right) \tag{19}$$

Using these, we can obtain the Fano factor of m, $FF\left(\widetilde{R_T}\right)$, defined as $\frac{\langle m^2\rangle-\langle m\rangle^2}{\langle m\rangle}$ (Supplementary Table 2) as follows:

$$FF\left(\widetilde{R_T}\right)=1+\frac{\tilde{\alpha}\left[\left(\widetilde{R_A}(\widetilde{R_T},\widetilde{K_s})+\widetilde{K_b}+\widetilde{K_d}+\tilde{\beta}\right)+\left(1+\widetilde{K_d}+\tilde{\beta}\right)\tilde{J}(\widetilde{R_T},\widetilde{K_a},\widetilde{K_s})\frac{\tilde{A}(\widetilde{R_T},\widetilde{K_s})}{\widetilde{K_a}}\frac{\tilde{R}(\widetilde{R_T},\widetilde{K_s})}{\widetilde{K_b}}\right]}{\left(1+\tilde{I}(\widetilde{R_T},\widetilde{K_a},\widetilde{K_s})\frac{\tilde{A}(\widetilde{R_T},\widetilde{K_s})}{\widetilde{K_a}}+\tilde{J}(\widetilde{R_T},\widetilde{K_a},\widetilde{K_s})\frac{\tilde{A}(\widetilde{R_T},\widetilde{K_s})}{\widetilde{K_a}}\frac{\tilde{R}(\widetilde{R_T},\widetilde{K_s})}{\widetilde{K_b}}\right)\left[\left(\widetilde{K_b}+\widetilde{K_d}+\tilde{\beta}\right)\left(\tilde{A}(\widetilde{R_T},\widetilde{K_s})+\widetilde{K_a}+\tilde{\beta}\right)+\left(\widetilde{K_b}+\widetilde{K_a}+\tilde{\beta}\right)\widetilde{R_A}(\widetilde{R_T},\widetilde{K_s})+\left(1+\widetilde{K_d}+\tilde{\beta}\right)\tilde{R}(\widetilde{R_T},\widetilde{K_s})\right]}.$$

Supplementary Table 1. Propensity functions of reactions and parameter values for all models.

Model	Reaction	Propensity function	Parameter value
The cooperative binding-based switch	$E_{000} \to E_{000} + M$	$\tilde{\alpha} n_{E_{000}}$	$\tilde{\alpha} = 10^0$<br>$\tilde{\beta} = 10^{-2}$<br>$c = 10^{-4}$
	$E_{001} \to E_{001} + M$	$\tilde{\alpha} n_{E_{001}}$	
	$E_{010} \to E_{010} + M$	$\tilde{\alpha} n_{E_{010}}$	
	$E_{100} \to E_{100} + M$	$\tilde{\alpha} n_{E_{100}}$	
	$E_{011} \to E_{011} + M$	$\tilde{\alpha} n_{E_{011}}$	
	$E_{101} \to E_{101} + M$	$\tilde{\alpha} n_{E_{101}}$	
	$E_{110} \to E_{110} + M$	$\tilde{\alpha} n_{E_{110}}$	
	$M \to \phi$	$\tilde{\beta} n_M$	
	$E_{000} \to E_{001}$	$n_{E_{000}}$	
	$E_{001} \to E_{000}$	$n_{E_{001}} / \widetilde{R_T}$	
	$E_{000} \to E_{010}$	$n_{E_{000}}$	
	$E_{010} \to E_{000}$	$n_{E_{010}} / \widetilde{R_T}$	
	$E_{000} \to E_{100}$	$n_{E_{000}}$	
	$E_{100} \to E_{000}$	$n_{E_{100}} / \widetilde{R_T}$	
	$E_{001} \to E_{011}$	$n_{E_{001}}$	
	$E_{011} \to E_{001}$	$c n_{E_{011}} / \widetilde{R_T}$	
	$E_{001} \to E_{101}$	$n_{E_{001}}$	
	$E_{101} \to E_{001}$	$c n_{E_{101}} / \widetilde{R_T}$	
	$E_{010} \to E_{011}$	$n_{E_{010}}$	
	$E_{011} \to E_{010}$	$c n_{E_{011}} / \widetilde{R_T}$	
	$E_{010} \to E_{110}$	$n_{E_{010}}$	
	$E_{110} \to E_{010}$	$c n_{E_{110}} / \widetilde{R_T}$	
	$E_{100} \to E_{101}$	$n_{E_{100}}$	
	$E_{101} \to E_{100}$	$c n_{E_{101}} / \widetilde{R_T}$	
	$E_{100} \to E_{110}$	$n_{E_{100}}$	
	$E_{110} \to E_{100}$	$c n_{E_{110}} / \widetilde{R_T}$	
	$E_{011} \to E_{111}$	$n_{E_{011}}$	

	$E_{111} \rightarrow E_{011}$	$c^2 n_{E_{111}}/\widetilde{R_T}$	
	$E_{101} \rightarrow E_{111}$	$n_{E_{101}}$	
	$E_{111} \rightarrow E_{101}$	$c^2 n_{E_{111}}/\widetilde{R_T}$	
	$E_{110} \rightarrow E_{111}$	$n_{E_{110}}$	
	$E_{111} \rightarrow E_{110}$	$c^2 n_{E_{111}}/\widetilde{R_T}$	
The sequestration-based switch	$E_A \rightarrow E_A + M$	$\tilde{\alpha} n_{E_A}$	$\tilde{\alpha} = 10^0$<br>$\tilde{\beta} = 10^{-2}$<br>$\widetilde{K_a} = 10^{-3}$<br>$\widetilde{K_s} = 10^{-5}$
	$M \rightarrow \phi$	$\tilde{\beta} n_M$	
	$E_F \rightarrow E_A$	$\tilde{A}(\widetilde{R_T}, \widetilde{K_s})$	
	$E_A \rightarrow E_F$	$\widetilde{K_a}$	
The sequestration- and blocking-based switch	$E_A \rightarrow E_A + M$	$\tilde{\alpha} n_{E_A}$	$\tilde{\alpha} = 10^0$<br>$\tilde{\beta} = 10^{-2}$<br>$\widetilde{K_a} = 10^{-3}$<br>$\widetilde{K_s} = 10^{-5}$<br>$\widetilde{K_b} = 10^{-3}$ *
	$M \rightarrow \phi$	$\tilde{\beta} n_M$	
	$E_F \rightarrow E_A$	$\tilde{A}(\widetilde{R_T}, \widetilde{K_s}) n_{E_F}$	
	$E_A \rightarrow E_F$	$\widetilde{K_a} n_{E_A}$	
	$E_A \rightarrow E_R$	$\tilde{R}(\widetilde{R_T}, \widetilde{K_s}) n_{E_A}$	
	$E_R \rightarrow E_A$	$\widetilde{K_b} n_{E_R}$	
The sequestration-, blocking-, and displacement-based switch	$E_A \rightarrow E_A + M$	$\tilde{\alpha} n_{E_A}$	$\tilde{\alpha} = 10^0$<br>$\tilde{\beta} = 10^{-2}$<br>$\widetilde{K_a} = 10^{-3}$<br>$\widetilde{K_s} = 10^{-5}$<br>$\widetilde{K_b} = 10^{-3}$ *<br>$\widetilde{K_d} = 10^1$
	$M \rightarrow \phi$	$\tilde{\beta} n_M$	
	$E_F \rightarrow E_A$	$\tilde{A}(\widetilde{R_T}, \widetilde{K_s}) n_{E_F}$	
	$E_A \rightarrow E_F$	$\widetilde{K_a} n_{E_A}$	
	$E_A \rightarrow E_R$	$\tilde{R}(\widetilde{R_T}, \widetilde{K_s}) n_{E_A}$	
	$E_R \rightarrow E_A$	$\widetilde{K_b} n_{E_R}$	
	$E_F \rightarrow E_R$	$\widetilde{R_A}(\widetilde{R_T}, \widetilde{K_s}) n_{E_F}$	
	$E_R \rightarrow E_F$	$\widetilde{K_d} n_{E_R}$	

Here, n_X is the number of X and $\tilde{A}(\widetilde{R_T}, \widetilde{K_s}) = \frac{1-\widetilde{R_T}-\widetilde{K_s}+\sqrt{(1-\widetilde{R_T}-\widetilde{K_s})^2+4\widetilde{K_s}}}{2}$, $\tilde{R}(\widetilde{R_T}, \widetilde{K_s}) = \frac{\widetilde{R_T}-1-\widetilde{K_s}+\sqrt{(1-\widetilde{R_T}-\widetilde{K_s})^2+4\widetilde{K_s}}}{2}$, and $\widetilde{R_A}(\widetilde{R_T}, \widetilde{K_s}) = \frac{1+\widetilde{R_T}+\widetilde{K_s}-\sqrt{(1-\widetilde{R_T}-\widetilde{K_s})^2+4\widetilde{K_s}}}{2}$.

$*$ $\widetilde{K_b} = 10^{-1}$ is used in Supplementary Fig. 2.

Supplementary Table 2. The transcriptional activity and the Fano factor for all models.

The cooperative binding-based switch	Transcriptional activity	$1-\frac{\widetilde{R_T}^3}{c^3+3c^3\widetilde{R_T}+3c^2\widetilde{R_T}^2+\widetilde{R_T}^3}$
	Fano factor	$1+\frac{\tilde{\alpha}\widetilde{R_T}^4\left[3(\tilde{\beta}+2)(\tilde{\beta}+3)\widetilde{R_T}^4+\left[3c\tilde{\beta}^2+(24c+3)\tilde{\beta}+45c\right]\widetilde{R_T}^3+\left[c\tilde{\beta}^2+3c(2c+3)\tilde{\beta}+2c(9c+10)\right]\widetilde{R_T}^2+c\left[(1+2c)\tilde{\beta}+(1+12c)\right]\widetilde{R_T}+2c^2\right]}{\begin{array}{c}3(\tilde{\beta}+1)(\tilde{\beta}+2)(\tilde{\beta}+3)\widetilde{R_T}^8+3\left[c(3c+1)\tilde{\beta}^3+(21c^2+8c+1)\tilde{\beta}^2+(48c^2+17c+1)\tilde{\beta}+6c(1+6c)\right]\widetilde{R_T}^7 \\ +c\left[6(1+36c^2+27c^3)+(20+36c+315c^2+135c^3)\tilde{\beta}+3(3+5c+45c^2+9c^3)\tilde{\beta}^2+(1+18c^2)\tilde{\beta}^3\right]\widetilde{R_T}^6 \\ +c\left[3c^2(3c+2)\tilde{\beta}^3+(54c^4+90c^3+57c^2+2c+1)\tilde{\beta}^2+(324c^4+252c^3+126c^2+12c+1)\tilde{\beta}+18c^2(27c^2+6c+4)\right]\widetilde{R_T}^5 \\ +c^2\left[6c^2\tilde{\beta}^3+3c(9c^3+12c^2+19c+2)\tilde{\beta}^2+(243c^4+198c^3+153c^2+9c+2)\tilde{\beta}+18c^2(27c^2+9c+4)\right]\widetilde{R_T}^4 \\ +c^4\left[\tilde{\beta}^3+6(3c^2+2c+2)\tilde{\beta}^2+(54c^3+153c^2+72c+29)\tilde{\beta}+6(27c^3+54c^2+2)\right]\widetilde{R_T}^3 \\ +c^4\left[(3c^2+2c+1)\tilde{\beta}^2+(36c^3+33c^2+18c+1)\tilde{\beta}+54c^2(1+3c)\right]\widetilde{R_T}^2+c^5\left[(6c^2+3c+2)\tilde{\beta}+54c^2\right]\widetilde{R_T}+6c^7\end{array}}$
The sequestration-based switch	Transcriptional activity	$\frac{\frac{\tilde{A}(\widetilde{R_T},\widetilde{K_S})}{\widetilde{K_a}}}{1+\frac{\tilde{A}(\widetilde{R_T},\widetilde{K_S})}{\widetilde{K_a}}}$, where $\tilde{A}\left(\widetilde{R_T},\widetilde{K_s}\right)=\frac{1-\widetilde{R_T}-\widetilde{K_s}+\sqrt{(1-\widetilde{R_T}-\widetilde{K_s})^2+4\widetilde{K_s}}}{2}$.
	Fano factor	$1+\frac{\tilde{\alpha}}{\left(1+\frac{\tilde{A}\left(\widetilde{R_T},\widetilde{K_s}\right)}{\widetilde{K_a}}\right)\left(\tilde{A}\left(\widetilde{R_T},\widetilde{K_s}\right)+\widetilde{K_a}+\tilde{\beta}\right)}$
The sequestration- and blocking-based switch	Transcriptional activity	$\frac{\frac{\tilde{A}(\widetilde{R_T},\widetilde{K_S})}{\widetilde{K_a}}}{1+\frac{\tilde{A}(\widetilde{R_T},\widetilde{K_S})}{\widetilde{K_a}}+\frac{\tilde{A}(\widetilde{R_T},\widetilde{K_S})}{\widetilde{K_a}}\frac{\tilde{R}(\widetilde{R_T},\widetilde{K_S})}{\widetilde{K_b}}}$, where $\tilde{R}\left(\widetilde{R_T},\widetilde{K_s}\right)=\frac{\widetilde{R_T}-1-\widetilde{K_s}+\sqrt{(1-\widetilde{R_T}-\widetilde{K_s})^2+4\widetilde{K_s}}}{2}$.
	Fano factor	$1+\frac{\tilde{\alpha}\left[\widetilde{K_b}+\tilde{\beta}+\left(\tilde{A}\left(\widetilde{R_T},\widetilde{K_s}\right)+\tilde{\beta}\right)\frac{\tilde{A}\left(\widetilde{R_T},\widetilde{K_s}\right)}{\widetilde{K_a}}\frac{\tilde{R}\left(\widetilde{R_T},\widetilde{K_s}\right)}{\widetilde{K_b}}\right]}{\left(1+\frac{\tilde{A}\left(\widetilde{R_T},\widetilde{K_s}\right)}{\widetilde{K_a}}+\frac{\tilde{A}\left(\widetilde{R_T},\widetilde{K_s}\right)}{\widetilde{K_a}}\frac{\tilde{R}\left(\widetilde{R_T},\widetilde{K_s}\right)}{\widetilde{K_b}}\right)\left[\widetilde{K_a}\left(\widetilde{K_b}+\tilde{\beta}\right)+\left(\tilde{A}\left(\widetilde{R_T},\widetilde{K_s}\right)+\tilde{\beta}\right)\left(\tilde{R}\left(\widetilde{R_T},\widetilde{K_s}\right)+\widetilde{K_b}+\tilde{\beta}\right)\right]}$
The sequestration-, blocking- and displacement-based switch	Transcriptional activity	$\frac{\tilde{I}\left(\widetilde{R_T},\widetilde{K_a},\widetilde{K_s}\right)\frac{\tilde{A}\left(\widetilde{R_T},\widetilde{K_s}\right)}{\widetilde{K_a}}}{1+\tilde{I}\left(\widetilde{R_T},\widetilde{K_a},\widetilde{K_s}\right)\frac{\tilde{A}\left(\widetilde{R_T},\widetilde{K_s}\right)}{\widetilde{K_a}}+\tilde{J}\left(\widetilde{R_T},\widetilde{K_a},\widetilde{K_s}\right)\frac{\tilde{A}\left(\widetilde{R_T},\widetilde{K_s}\right)}{\widetilde{K_a}}\frac{\tilde{R}\left(\widetilde{R_T},\widetilde{K_s}\right)}{\widetilde{K_b}}}$,<br>where $\tilde{I}\left(\widetilde{R_T},\widetilde{K_a},\widetilde{K_s}\right)=\frac{\widetilde{K_s}+\sigma\widetilde{K_a}+\tilde{R}(\widetilde{R_T},\widetilde{K_s})}{\widetilde{K_s}+\sigma\widetilde{K_a}+\sigma\tilde{R}(\widetilde{R_T},\widetilde{K_s})}$, $\tilde{J}\left(\widetilde{R_T},\widetilde{K_a},\widetilde{K_s}\right)=\frac{\widetilde{K_s}+\sigma\widetilde{K_a}+\sigma\tilde{R}(\widetilde{R_T},\widetilde{K_s})}{\widetilde{K_s}+\sigma\widetilde{K_a}+\sigma\tilde{R}(\widetilde{R_T},\widetilde{K_s})}$, $\sigma=\frac{\widetilde{K_s}\widetilde{K_d}}{\widetilde{K_a}\widetilde{K_b}}$.
	Fano factor	$1+\frac{\tilde{\alpha}\left[\left(\widetilde{R_A}(\widetilde{R_T},\widetilde{K_s})+\widetilde{K_b}+\widetilde{K_d}+\tilde{\beta}\right)+\left(1+\widetilde{K_d}+\tilde{\beta}\right)\tilde{J}(\widetilde{R_T},\widetilde{K_a},\widetilde{K_s})\frac{\tilde{A}(\widetilde{R_T},\widetilde{K_s})}{\widetilde{K_a}}\frac{\tilde{R}(\widetilde{R_T},\widetilde{K_s})}{\widetilde{K_b}}\right]}{\left(1+\tilde{I}(\widetilde{R_T},\widetilde{K_a},\widetilde{K_s})\frac{\tilde{A}(\widetilde{R_T},\widetilde{K_s})}{\widetilde{K_a}}+\tilde{J}(\widetilde{R_T},\widetilde{K_a},\widetilde{K_s})\frac{\tilde{A}(\widetilde{R_T},\widetilde{K_s})}{\widetilde{K_a}}\frac{\tilde{R}(\widetilde{R_T},\widetilde{K_s})}{\widetilde{K_b}}\right)\left[\left(\widetilde{K_b}+\widetilde{K_d}+\tilde{\beta}\right)\left(\tilde{A}(\widetilde{R_T},\widetilde{K_s})+\widetilde{K_a}+\tilde{\beta}\right)+\left(\widetilde{K_b}+\widetilde{K_a}+\tilde{\beta}\right)\widetilde{R_A}(\widetilde{R_T},\widetilde{K_s})+\left(1+\widetilde{K_d}+\tilde{\beta}\right)\tilde{R}(\widetilde{R_T},\widetilde{K_s})\right]}$

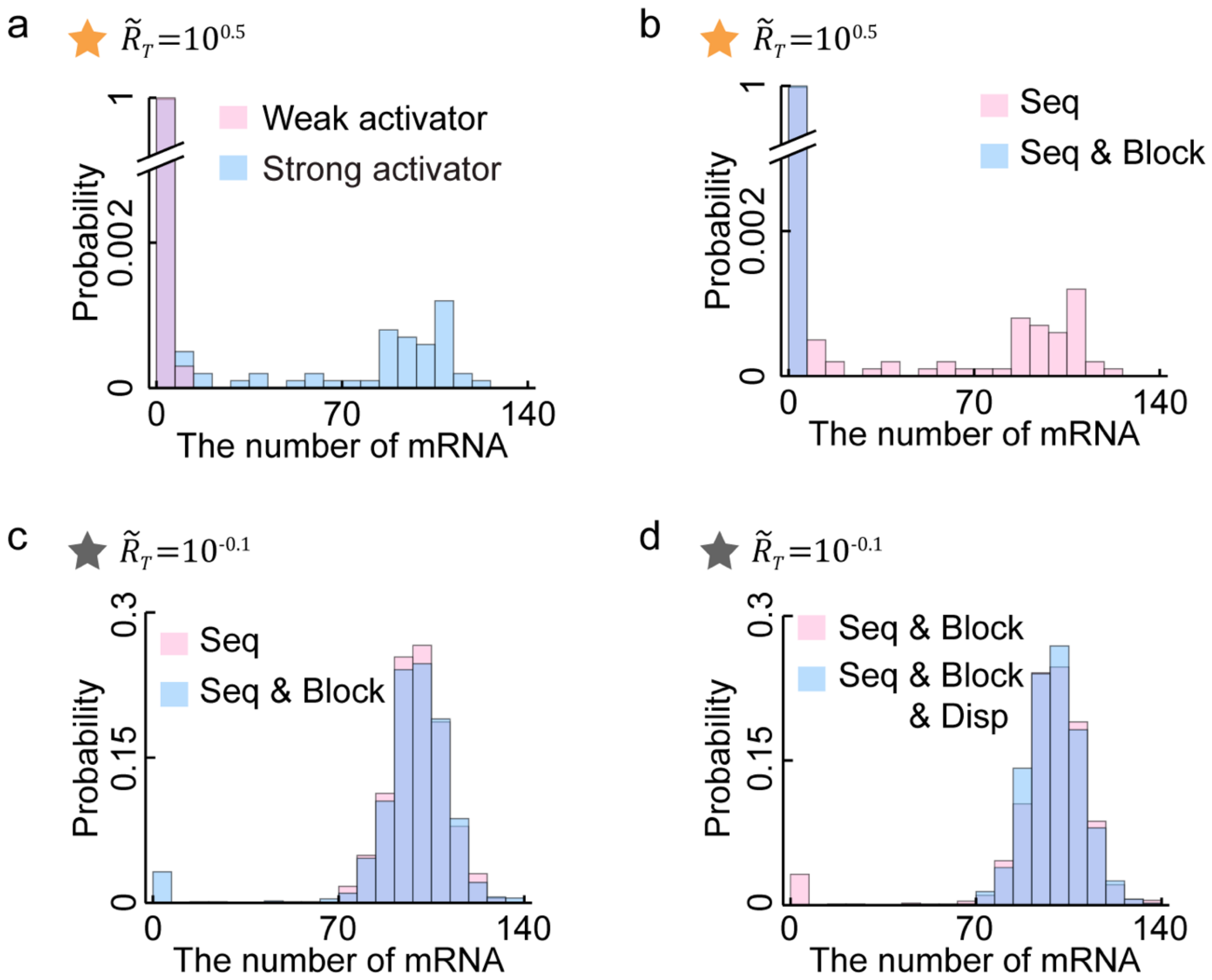

Supplementary Figure 1. The stationary distributions of mRNAs transcribed during TRP and TAP in each transcriptional ultrasensitive switch. a In the sequestration-based switch, the stationary distribution of mRNAs shows an additional peak far from zero, leading to bimodality with a strong activator (blue bars) unlike with a weak activator (red bars) during TRP. **b** In contrast, in the sequestration- and blocking-based switch, the stationary distribution of mRNAs is concentrated around zero (blue bars) without bimodality observed in sole sequestration (red bars). **c** However, during TAP, it shows an additional peak around zero, leading to bimodality with blocking (blue bars) unlike with sequestration alone (red bars). **d** In the sequestration-, blocking-, and displacement-based switch, such an additional peak around zero is removed from the stationary distribution of mRNAs during TAP.

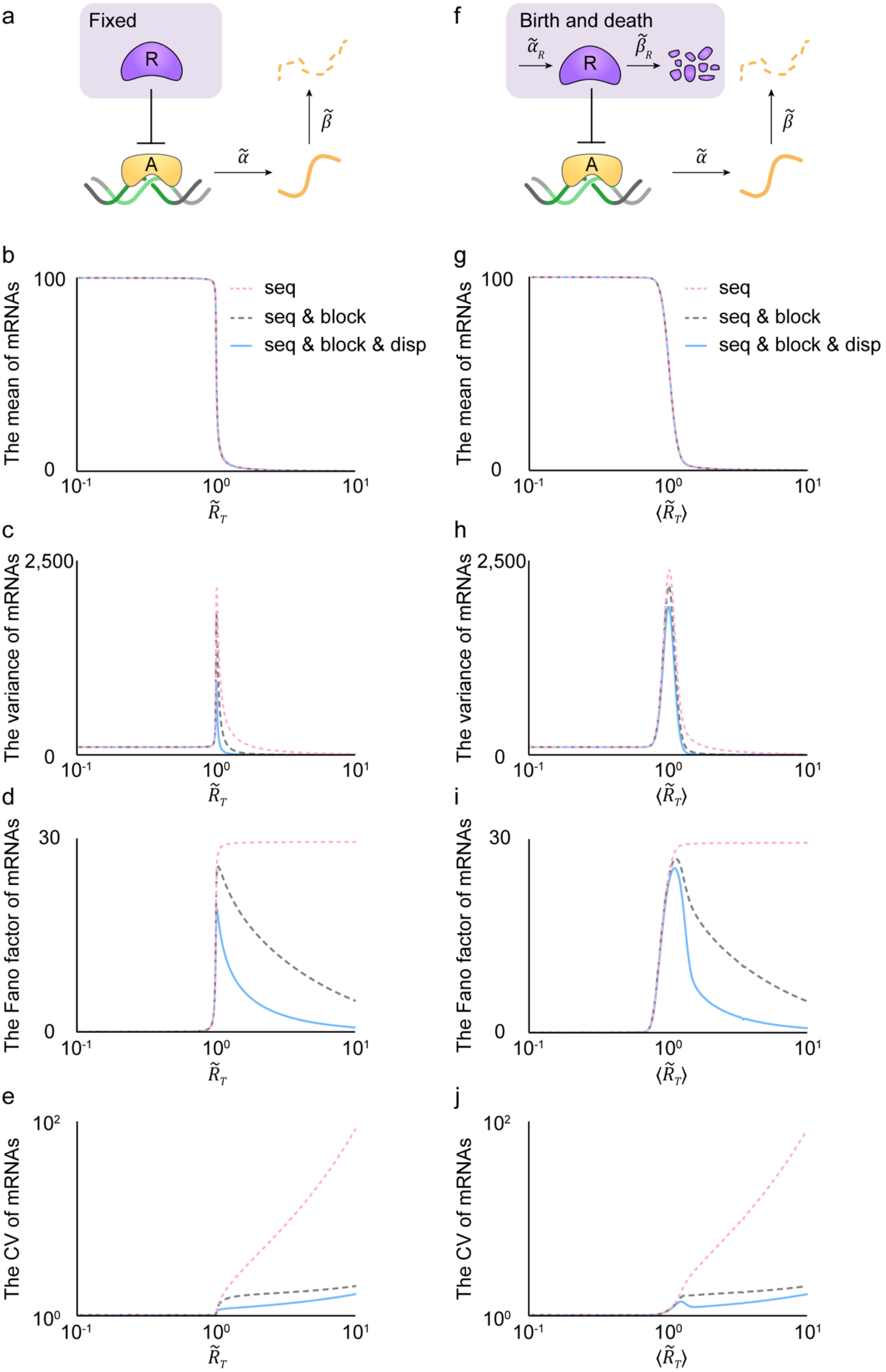

a
Fixed
R
A
$\tilde{\alpha}$
$\tilde{\beta}$
f
Birth and death
$\tilde{\alpha}_R$
$\tilde{\beta}_R$
b
g
The mean of mRNAs
seq
seq & block
seq & block & disp
c
h
The variance of mRNAs
2,500
d
i
The Fano factor of mRNAs
30
e
j
The CV of mRNAs
10^2
10^0
10^{-1}
10^1
$\tilde{R}_T$
$\langle\tilde{R}_T\rangle$

Supplementary Figure 2. The sequestration, blocking, and displacement-based switch exhibits superior robustness to noise compared to the other two types of switches, even when the means of the mRNAs are identical and the total number of repressors fluctuates. a In the three types of transcriptional ultrasensitive switches used in Figs. 2-4, the number of the total repressors (R_T) is fixed. **b** These switches can exhibit the same mean of mRNAs with respect to R_T by modifying the value of $\widetilde{K_b}$ (Supplementary Table 1), resulting in the same sensitivity. **c** On the other hand, the switch utilizing sequestration, blocking, and displacement shows the lowest variance in the number of mRNAs among the three types of switches. **d, e** Consequently, both the Fano factor (**d**) and the coefficient of variation (CV), defined as the standard deviation over the mean (**e**), are also lower for the triple-mechanism switch than for the others. **f** When birth-death reactions of $\mathrm{R_T}$ with rates $\widetilde{\alpha_R}$ and $\widetilde{\beta_R}$ are incorporated into the ultrasensitive switches, R_T fluctuates rather than remains fixed. **g, h** As the dynamics of R_T follow a birth-death process governed by the Poisson distribution, the mean molar ratio ($\langle\widetilde{R_T}\rangle$) is $(\widetilde{\alpha_R}/\widetilde{\beta_R})/A_T$. Then, depending on the mean molar ratio, we calculated how the mean ($\mathrm{E[n_M]}$) and variance ($Var(n_M)$) of mRNAs change. Specifically, by using the law of total expectation and variance, we can calculate $E[n_M] = E\big[E[n_M|R_T]\big]$ and $Var(n_M) = E[Var(n_M|R_T)] + Var(E[n_M|R_T])$ because the conditional mean and variance can be calculated: $E[n_M|R_T] = \frac{\tilde{\alpha}}{\tilde{\beta}}TA(R_T/A_T)$, $Var(n_M|R_T) = E[n_M|R_T]FF(R_T/A_T)$, where $TA(\cdot)$ and $FF(\cdot)$ are the equations for the transcriptional activity and the Fano factor (Supplementary Table 2). As a result, $E[n_M] = \int_0^\infty \frac{\tilde{\alpha}}{\tilde{\beta}}TA(R_T/A_T)\,Poisson\big(R_T;\widetilde{\alpha_R}/\widetilde{\beta_R}\big)dR_T$ and $Var(n_M) = \int_0^\infty \frac{\tilde{\alpha}}{\tilde{\beta}}TA(R_T/A_T)FF(R_T/A_T)\,Poisson\big(R_T;\widetilde{\alpha_R}/\widetilde{\beta_R}\big)dR_T + \int_0^\infty \left[\frac{\tilde{\alpha}}{\tilde{\beta}}TA(R_T/A_T)\right]^2 Poisson\big(R_T;\widetilde{\alpha_R}/\widetilde{\beta_R}\big)dR_T - \left(\int_0^\infty \frac{\tilde{\alpha}}{\tilde{\beta}}TA(R_T/A_T)\,Poisson\big(R_T;\widetilde{\alpha_R}/\widetilde{\beta_R}\big)dR_T\right)^2$. Here, $Poisson\big(R_T;\widetilde{\alpha_R}/\widetilde{\beta_R}\big) = \frac{(\widetilde{\alpha_R}/\widetilde{\beta_R})^{R_T}\,e^{-\widetilde{\alpha_R}/\widetilde{\beta_R}}}{R_T!}$ represents the probability density function of the Poisson distribution with the mean of $\widetilde{\alpha_R}/\widetilde{\beta_R}$. Then, the calculated mean of mRNAs is identical in the three types of switches (**g**). On the other hand, the switch utilizing sequestration, blocking, and displacement shows the least variance of mRNAs among the three types of switches (**h**). **i, j** As a result, the Fano factor (**i**) and CV (**j**) are also lower for the triple-mechanism switch than for the others. Here, $\widetilde{\beta_R} = 10^{-4}$ and $A_T = 100$ are used, and $\widetilde{\alpha_R}$ is adjusted to vary the value of $\langle\widetilde{R_T}\rangle$.

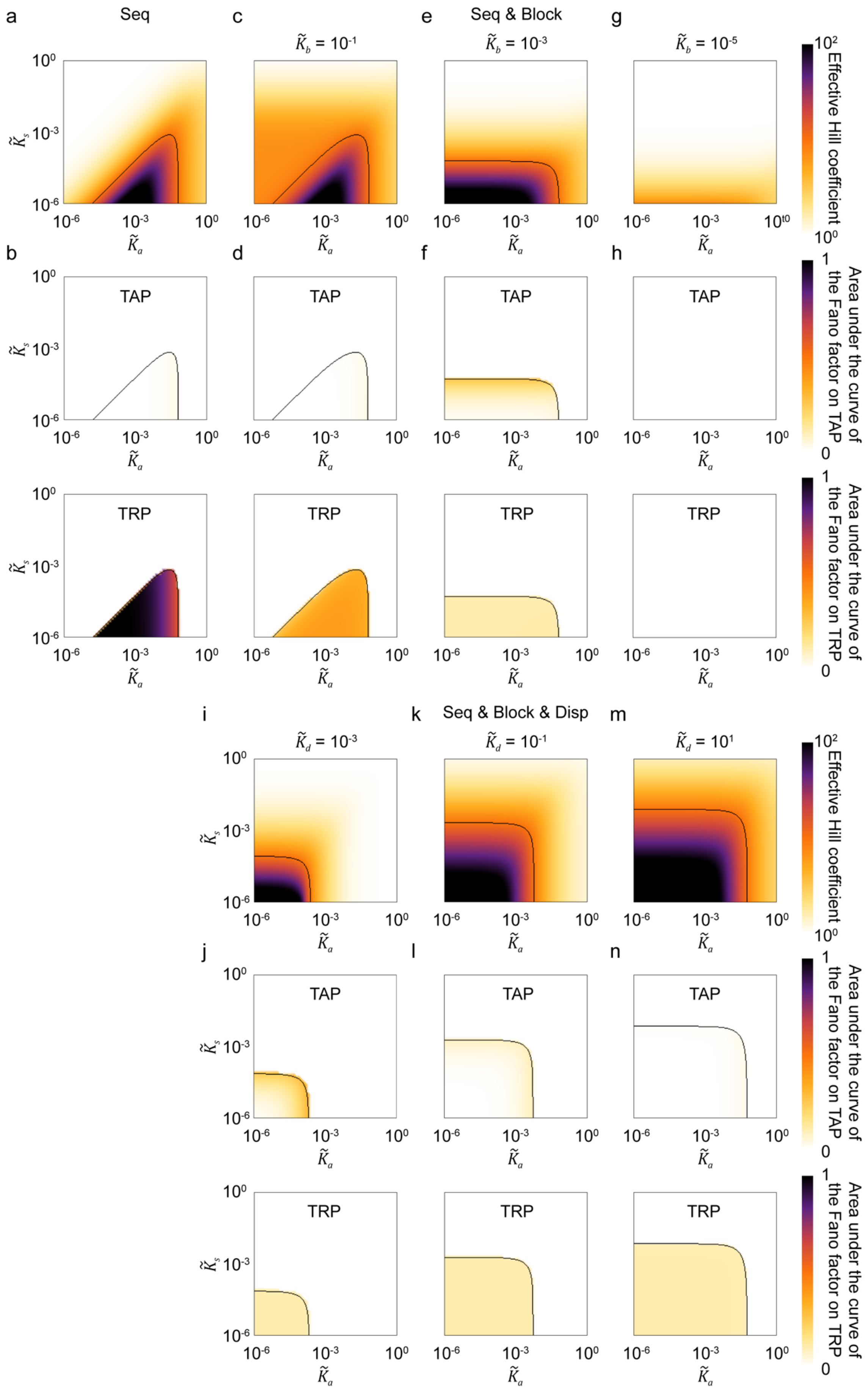

a
Seq
c
$\tilde{K}_b$ = 10^{-1}
e
Seq & Block
$\tilde{K}_b$ = 10^{-3}
g
$\tilde{K}_b$ = 10^{-5}
Effective Hill coefficient
10^2
10^0
b
d
f
h
TAP
TRP
Area under the curve of the Fano factor on TAP
Area under the curve of the Fano factor on TRP
1
0
$\tilde{K}_s$
$\tilde{K}_a$
10^0
10^{-3}
10^{-6}
i
$\tilde{K}_d$ = 10^{-3}
k
Seq & Block & Disp
$\tilde{K}_d$ = 10^{-1}
m
$\tilde{K}_d$ = 10^1
j
l
n

Supplementary Figure 3. The transcriptional switch utilizing all three mechanisms – sequestration, blocking, and displacement – can generate ultrasenstivity robust to noise. a In the sole sequestration-based switch, strong sequestration and strong activators (i.e., $\widetilde{K_s} < \widetilde{K_a}$ and $\widetilde{K_a} < 10^{-1}$) are required to generate ultrasensitive transcriptional activity. The sensitivity of the transcriptional activity is quantified using the effective Hill coefficient. The black line denotes the effective Hill coefficient of 10. **b** When the effective Hill coefficient exceeds 10 (i.e., when ultrasensitivity is generated; below the black line), transcriptional noise is low during TRP (top), but high during TAP (bottom). Transcriptional noise is quantified by the normalized area of the plot for the Fano factor with respect to $\widetilde{R_T}$ (e.g., Fig. 2b(ii)) on $[10^{-1}, 10^{-0.1}]$ and $[10^{0.1}, 10^{1}]$ during TRP and TAP, respectively). **c** With the addition of weak blocking ($\widetilde{K_b} = 10^{-1}$) to the sole sequestration-based switch, the sensitivity remains comparable to that generated by the sole sequestration-based switch. **d** Concurrently, the transcriptional noise during TRP is reduced (bottom). **e-f** Stronger blocking ($\widetilde{K_b} = 10^{-3}$) further diminishes transcriptional noise during TRP (f, bottom), but elevates it during TAP (f, top). **g-h** When the blocking becomes too strong ($\widetilde{K_b} = 10^{-5}$), ultrasensitivity is not generated. **i** After the addition of weak displacement ($\widetilde{K_d} = 10^{-3}$) to the sequestration- and blocking-based switch with $\widetilde{K_b} = 10^{-3}$, ultrasensitivity is generated within a narrower range. **j** In this range, the transcriptional noise during TAP is slightly higher (top), while during TRP it remains comparable (bottom) to that generated by the sequestration- and blocking-based switch (f). **k-l** Stronger displacement ($\widetilde{K_d} = 10^{-1}$) can generate ultrasensitivity over a broader range (k), while reducing the transcriptional noise during TAP (l, top) compared to the sequestration- and blocking-based switch (f). **m-n** Moreover, as further stronger displacement ($\widetilde{K_d} = 10^{1}$) is added (m), ultrasensitivity is generated over a broader range and the transcriptional noise is more reduced during TAP (n, top).

Supplementary References

1. Sanchez, A., Garcia, H.G., Jones, D., Phillips, R. and Kondev, J. (2011) Effect of promoter architecture on the cell-to-cell variability in gene expression. *PLoS Comput Biol*, **7**, e1001100.
2. Song, Y.M., Hong, H. and Kim, J.K. (2021) Universally valid reduction of multiscale stochastic biochemical systems using simple non-elementary propensities. *PLoS Comput Biol*, **17**, e1008952.
3. Kim, J.K., Josic, K. and Bennett, M.R. (2014) The validity of quasi-steady-state approximations in discrete stochastic simulations. *Biophys J*, **107**, 783-793.
4. Kim, J.K., Josic, K. and Bennett, M.R. (2015) The relationship between stochastic and deterministic quasi-steady state approximations. *Bmc Syst Biol*, **9**.
5. Kim, J.K. and Sontag, E.D. (2017) Reduction of multiscale stochastic biochemical reaction networks using exact moment derivation. *PLoS Comput Biol*, **13**, e1005571.
